# Food for Pollinators: Quantifying the Nectar and Pollen Resources of Urban Flower Meadows

**DOI:** 10.1371/journal.pone.0158117

**Published:** 2016-06-24

**Authors:** Damien M. Hicks, Pierre Ouvrard, Katherine C. R. Baldock, Mathilde Baude, Mark A. Goddard, William E. Kunin, Nadine Mitschunas, Jane Memmott, Helen Morse, Maria Nikolitsi, Lynne M. Osgathorpe, Simon G. Potts, Kirsty M. Robertson, Anna V. Scott, Frazer Sinclair, Duncan B. Westbury, Graham N. Stone

**Affiliations:** 1 Institute of Evolutionary Biology, University of Edinburgh, Kings Buildings, Charlotte Auerbach Road, Edinburgh EH9 3JT, United Kingdom; 2 Earth and Life Institute - Agronomy, Université catholique de Louvain, Place Croix du Sud 2, 1348 Louvain-la-Neuve, Belgium; 3 School of Biological Sciences, University of Bristol, 24 Tyndall Avenue, Bristol, BS8 1TQUG, United Kingdom; 4 Cabot Institute, University of Bristol, Woodland Road, Bristol, BS8 1UJ, United Kingdom; 5 Collegium Sciences et Techniques, EA 1207 LBLGC, Université d’Orléans, 45067, Orléans, France; 6 School of Biology, University of Leeds, Leeds, LS2 9JT, United Kingdom; 7 Centre for Agri-Environmental Research, School of Agriculture, Policy and Development, University of Reading, Reading, RG6 6AR, United Kingdom; 8 Royal Society for the Protection of Birds, Gola Rainforest National Park, Kenema, Sierra Leone; 9 Institute of Science & the Environment, The University of Worcester, Henwick Grove, Worcester, WR2 6AJ, United Kingdom; Institute of Botany, CHINA

## Abstract

Planted meadows are increasingly used to improve the biodiversity and aesthetic amenity value of urban areas. Although many ‘pollinator-friendly’ seed mixes are available, the floral resources these provide to flower-visiting insects, and how these change through time, are largely unknown. Such data are necessary to compare the resources provided by alternative meadow seed mixes to each other and to other flowering habitats. We used quantitative surveys of over 2 million flowers to estimate the nectar and pollen resources offered by two exemplar commercial seed mixes (one annual, one perennial) and associated weeds grown as 300m^2^ meadows across four UK cities, sampled at six time points between May and September 2013. Nectar sugar and pollen rewards per flower varied widely across 65 species surveyed, with native British weed species (including dandelion, *Taraxacum* agg.) contributing the top five nectar producers and two of the top ten pollen producers. Seed mix species yielding the highest rewards per flower included *Leontodon hispidus*, *Centaurea cyanus* and *C*. *nigra* for nectar, and *Papaver rhoeas*, *Eschscholzia californica* and *Malva moschata* for pollen. Perennial meadows produced up to 20x more nectar and up to 6x more pollen than annual meadows, which in turn produced far more than amenity grassland controls. Perennial meadows produced resources earlier in the year than annual meadows, but both seed mixes delivered very low resource levels early in the year and these were provided almost entirely by native weeds. Pollen volume per flower is well predicted statistically by floral morphology, and nectar sugar mass and pollen volume per unit area are correlated with flower counts, raising the possibility that resource levels can be estimated for species or habitats where they cannot be measured directly. Our approach does not incorporate resource quality information (for example, pollen protein or essential amino acid content), but can easily do so when suitable data exist. Our approach should inform the design of new seed mixes to ensure continuity in floral resource availability throughout the year, and to identify suitable species to fill resource gaps in established mixes.

## Introduction

There is increasing interest in the role of urban environments as habitats for wildlife, including species of conservation concern [1,2]. This interest stems from the fact that while natural habitats are declining and becoming increasingly fragmented in many parts of the world, urban habitats are expanding [2–6]. The current focus on pollinators is driven by the value of pollination services for supporting wider biodiversity and contributing to human food supplies (e.g. [7]), and growing evidence of diversity loss and range contractions for some species of wild pollinators in Europe and North America [8–10]. Studies to date (e.g. [11–17]) show that pollinator assemblages change along urbanisation gradients in a way that varies among pollinator taxa. While some urban environments support low pollinator diversity (e.g. [18]), others support high pollinator abundance and/or species richness [16,18–20]. A recent analysis of UK pollinator assemblages, for example, found cities to support higher bee species richness, but lower hoverfly abundance, than farmland [20]. Widespread public recognition of their ecological role has also made pollinators (and particularly bumblebees) an important flagship group for raising public awareness of human impacts on biodiversity [16].

Food limitation, resulting from decreasing flower diversity and quantity, is thought to be one of multiple causes for pollinator decline [21–24]. While dependence of pollinator populations on floral resources varies among taxa (see Discussion), the pollen harvested by bees is invested directly in the next generation [25–27]. Pollen from between 20 and several thousand flowers is required to rear a single solitary bee larva [22], and 120kg of nectar and 20kg of pollen are harvested annually by a single temperate European honeybee colony [28]. Growing numbers of studies have shown pollinator visitation to urban flowers [19,20,29–33] and a positive correlation between the abundances of flowers and pollinators [29,33–35]. These findings suggest that pollinator abundance and diversity could be increased by changes in urban land use that increase floral resource availability. Urban fruit and vegetable production is also probably dependent on local reservoirs of pollinators [30,36] whose life histories require sugar and protein at predictable points in the season.

Recent years have seen increased planting of seed mixes in urban landscapes, creating extensive flowering borders and urban meadows (e.g. [37,38]). Such ‘green infrastructure’ mixes can comprise both native and exotic species, and are often designed to contain primarily herbs rather than grasses (in contrast to natural hay meadows) [39,40]. Selected sets of species are also chosen to provide a long season with a high intensity of flowering and hence aesthetic impact [38,41]. The motivation for planting urban meadows commonly combines benefits for human quality of life [42,43] with desire to increase the biodiversity value of urban spaces. This is actively encouraged by a range of civil and planning awards and initiatives, including the UK’s Britain in Bloom (https://www.rhs.org.uk/communities/campaigns/britain-in-bloom/) and Green Flag Awards (http://www.greenflagaward.org/), and Belgium’s Plan Maya (http://biodiversite.wallonie.be/fr/plan-maya.html?IDC=5617). The expectation is that such flower-rich patches will provide more nectar and pollen resources for pollinators than the frequently mowed amenity grassland (i.e. cultivated open park grassland) that makes up many urban green spaces. There is evidence that pollinators visit urban flower plantings more than unplanted comparison plots [44], paralleling observations for flower margins planted adjacent to agricultural crops [45–48].

Numerous commercial annual and perennial seed mixes are available for establishing flower-rich habitat (see S5 Table). However, we do not know the nectar and pollen resources per flower provided by different seed mix species, or resource levels per unit area of different seed mixes. These are important knowledge gaps, because seed merchants cannot currently design (or landscape managers select) seed mixes on the basis of their ability to provide resources to pollinators. Seasonal timing (phenology), quality and quantity of floral resources are all important for pollinator populations [25,26,49]. In particular, seed mixes designed to support pollinators must deliver pollen and nectar throughout the season, without dips in seasonal resource availability that could potentially limit pollinator populations [50].

Given the wide range of available seed mixes, here we focus on quantifying the per-species nectar and pollen resources in two commercially available exemplars, one annual and one perennial, which show substantial overlap in species composition with available alternatives (S5 Table). The annual “Rainbow” mix supplied by Rigby Taylor is a market leader in the UK that contains 14 native and non-native annual species, and in 2014 and 2015 was planted over more than 100 hectares (ha) of urban meadows. The perennial “Special Pollen and Nectar Wildflower” mix sold by Emorsgate Seeds contains 23 native species and over the last six years has been sown on over 30 ha of parks and gardens and (in a slightly modified form), on *ca*. 120 ha of farmland, primarily for UK agri-environment schemes. The two mixes in our study include all of the species most commonly included in a panel of 10 commercially available pollinator seed mixes (S5 Table): *Centaurea cyanus*, *Leucanthemum vulgare* (5 mixes), *Centaurea nigra*, *Daucus carota*, *Lotus corniculatus*, *Silene dioica*, and *Trifolium pratense* (4 mixes), making them appropriate exemplars for study.

We planted each mix in replicate 300 m^2^ meadows in four UK towns and cities (Bristol, Edinburgh, Reading and Leeds) and compared the rewards provided by these meadows with control plots of amenity grassland. Because weed species such as *Taraxacum* agg. can provide significant resources to pollinators [51], we included non-mix (weed) species in surveys of all treatments. In this context, the weeds in our experiments comprise both native species and non-native garden-escape species, a common situation in urban environments. Our objectives are to (i) quantify the per-flower nectar and pollen resources provided by each seed mix and weed species, highlighting those providing high and low rewards; (ii) assess between-city variation in the composition of meadows resulting from planting the same mixes with the same protocols across the UK; and (iii) quantify meadow-level changes in floral resource provision through time and between meadow treatments. We explore the consequences of alternative sampling intensities for surveys of floral abundance and develop predictive statistical models for pollen volume per flower (based on floral morphology) and nectar and pollen resource per unit area (based on flower counts). Our approach does not incorporate variation in resource quality [52–54], but can easily be modified to do so where suitable data exist (see Discussion).

## Materials and Methods

### Seed mixes and experimental design

We used two commercially available flower seed mixes, one annual and the other perennial: Rigby Taylor’s ‘Rainbow Annual’ mix (14 species), and Emorsgate’s ‘EN1F Special Pollen and Nectar Wildflowers’ mix (23 species). Species compositions for these mixes, native/exotic status [55], and common names for all mix and weed species are given in S1 Table. These two mix types characterise the main alternatives available to landscape managers. Annual seed mixes provide a quick return by flowering in their first year, but they generally need to be replanted each year to maintain performance. Disturbance of the ground in the first year of planting a seed mix has the potential to trigger weed growth from the seed bank, which could result in increasing weed growth in meadows grown on the same site as bare soil is repeatedly exposed in subsequent years. We assess the significance of this effect here by comparing resource provision in annual meadows in their first year, and in the same seed mix replanted on the same site in a second year. Commercially available annual mixes often contain a mixture of native, naturalised and non-native species: examples in the mix we used include *Malcolmia maritima* native to Mediterranean Europe, and multiple American species (*Coreopsis* species, *Eschscholzia californica*, *Thelesperma burridgeanum* and *Cosmos bipinnatus*), included to provide a showy display throughout a long flowering season. In contrast, perennial seed mixes take longer to become established and can require additional labour costs for weeding in the year before they flower [38], but need replanting much less often. The seeds in the perennial mix we used were all sourced from native UK provenances.

Our experimental design comprised four treatments applied to amenity grassland in 80 sites across four UK towns and cities (Edinburgh, Leeds, Bristol, Reading, all termed cities hereafter), each having a total of 20 sites. In each city, we collected data in 2013 for five 300m^2^ replicates of each of the following four treatments: (i) annual mix sown in 2013 and sampled in its first year (A1); (ii) annual mix sown in 2012 and sampled after reseeding in 2013 in the same location (A2); (iii) perennial mix sown in 2012 and sampled in its second year in 2013, (iv) unplanted amenity grassland control, mown approximately every two weeks through the season, also sampled in 2013. Each treatment replicate had an area of 300 m^2^, with meadow shape varying among replicates as circumstances dictated. In each city, floral abundance in each treatment was surveyed at six time points between early May and early September 2013. Locations for all treatment replicates are detailed in S2 Table. To facilitate establishment of planted species and to maintain public acceptance of planted meadows, the largest and most visible weeds were removed at intervals. This included taxa (such as *Sonchus* species) subsequently shown to be highly rewarding, such that our resource estimates for weeds underestimate potential contributions from unweeded meadows. Large weed removal is nevertheless widely practised in urban green spaces, and our results are appropriate for such management.

### Quantification of floral resource per flower

We quantified floral resources per species in terms of daily nectar sugar mass and pollen volume [56] using protocols matching [57] for nectar and similar to those used in other studies [22,58]. We outline sampling methodologies below and provide detailed protocols in S2 and S3 Files. Floral resource measurements were made at the level of single flowers for all taxa except Asteraceae, for which resources were sampled at the level of the capitulum. For simplicity we use ‘flowers’ hereafter, and highlight contrasts between flowers and capitula where necessary. Grasses, sedges and wind-pollinated forbs were not sampled. Floral resource sampling for all species was carried out in Edinburgh in 2012 and 2013, using plants sampled as available across all 15 planted and five control replicate meadows. We recognise that our sampling thus does not incorporate environmental impacts on quantity or quality of resource provision by seeds of the same cultivars grown in different cities. To assess the extent to which our resource values agree with other estimates for the same species, we correlated our nectar resource data with values generated using the same methods recently published by Baude *et al*. [57].

#### (i) Nectar sampling

On days with no rain, nectar was allowed to accumulate for 24 hours in flowers from which insects were excluded by fine netting. Nectar quantification followed one of two protocols (see S2 File for detailed methods). When possible, nectar was collected directly using 1.0 μl microcapillaries (VWR International, UK) and the degrees Brix sugar content (g of sugar in 100g solution) estimated using a sucrose refractometer (VWR International, UK, using refractometers modified by the maker to accept very low volumes). Nectar sugar content of each sample (mg) was calculated from the equation *s* = 10*dvC*, where *v* is the volume of nectar (ml), and *d* is the density of a sucrose solution at a concentration *C* (g sucrose/100 g solution) as read on the refractometer [59,60]. The density was obtained as d = 0.0037921*C* + 0.0000178*C*^2^ + 0.9988603 [60].

We sampled 9.5 ± 0.6 (range 3–22) flowers per species (depending on availability) to estimate a species mean (see S1 Table). Where volumes of nectar were too small or concentrations of nectar too high to use this approach, nectar sugar was harvested by using a fine Gilson pipette to flush flowers with a known volume of distilled water and the Brix sugar content of the resulting solution estimated using a sucrose refractometer [57]. The mass of sugar contained was estimated as above. Nectar reward values are presented as mean sugar mass/floral unit ± 1 standard error of the mean. Nectar analyses covered 80% of species and over 99% of all flowers recorded in our surveys.

#### (ii) Pollen sampling

Pollen per flower was estimated for all species using flowers collected in Edinburgh treatment meadows in 2012 and 2013, for a total of 15,925 flowers across 64 species (see S3 File for detailed methods). Pollen was harvested using sonication (Dawe sonicleaner) from flowers that opened and dehisced in the lab. The collected pollen was suspended in 70% ethanol, and the number of pollen grains in a known volume aliquot counted on a haemocytometer slide. We estimated the volume of a sample of 100 pollen grains of each species using the formula for an ellipsoid (Volume = (4/3)*π*(A/2)*(B/2)^2^, where ‘a’ is the major axis and ‘b’ the minor axis of the pollen grain). Pollen volume per floral unit was then calculated as (total number of pollen grains/floral unit) x (mean volume per pollen grain). Sample size information for resource quantification in each species is provided in S1 Table, from a mean of 10.3 ± 0.6 (range 2–22) floral units/species, depending on availability. Pollen resource per floral unit per 24h was estimated by dividing the pollen volume per floral unit by floral longevity in days. This approach does not assume that pollen release is constant during the lifetime of each flower, but does assume that the proportion of flowers in the sampled population in each stage of pollen release (if it varies through floral life) was stable at the time longevity was measured (see below). Pollen resource values are presented as mean pollen volume/floral unit ± 1 standard error of the mean. Pollen resource per 24h was estimated for 78% of the species recorded in treatment plots, representing over 99% of the flowers present in our surveys (see S1 Table).

We explored the predictability of pollen volume per flower (or per floret for Asteraceae) from floral morphology, using a linear model incorporating anther size and number of stamens as predictors in the base R package [61]. To simplify use of the model, we allocated anther sizes for each species to one of four size classes (S3 Table): ‘tiny’ (for all Asteraceae), ‘small’ (e.g. *Galium verum*, *Stellaria media*; to a maximum anther volume of *ca*. 1.25mm^3^), ‘medium’ (e.g. *Ranunculus* spp., *Malcolmia maritima*; *ca*. 1.25–2.25mm^3^) and ‘large’ (e.g. *Papaver rhoeas*, *Chamerion angustifolium*: volume > 2.25mm^3^). Data on stamen numbers/floral unit are also provided in S3 Table. Normalising transformations for the parameters (log_e_ pollen per flower and 1/stamen number) were estimated by likelihood ratio tests using the powerTransform function in {car} [62], which maps variables to a family of functions to create a rank-preserving transformation of data using power functions. The regression model was fitted and checked for non-violation of assumptions of linearity, normality and homoscedasticity by inspection of residuals, fitted values and quantile plots.

We tested the predictive power of our model by estimating per-flower pollen volumes for a validation data set comprising 43 species quantified using the same lab protocol (Baude et al. unpublished data; see [57] for information on the Agriland Project generating these data). Species in the validation data set were selected to span the same range of measured pollen volumes. We attempted to develop similar predictive models for nectar sugar per flower based on the floral morphology traits of corolla diameter and corolla length. No useful predictive model resulted from this approach.

#### (iii) Floral longevity

Floral unit longevity was estimated by scoring the numbers of newly opening and newly closed (i.e. post-reproductive) flowers entering a fixed survey area (see S3 File for detailed methodology). Under the assumption that the population of flowers of a given species is approximately stable, floral longevity in days can be estimated as (2a+(b-c)) / ((b-c)d), where a = total open flowers on first observation, b = total newly-open flowers on second observation, c = total newly-closed flowers on second observation, and d = number of days between first and second observation. To meet the assumptions of this approach as closely as possible, species were observed in the middle of their flowering period. To minimise weather impacts on resources, observations were set up when the weather forecast was for no rain for the following day and the temperature was above 15°C. Sampling included the full range of flower ages, totalling approximately 50 flowers distributed on at least five plants. After marking the boundary of the study area and/or the inflorescences to be included, the area was surveyed on two occasions exactly 24 hours apart (or a multiple thereof for longer-lasting flowers). Longevity data for almost all species were collected for plants from Edinburgh meadows, with additional data where necessary from sites specified in S1 Table, sampled using the same protocol.

### Surveys of floral abundance and resources per meadow

We used quadrat surveys to quantify floral abundance in all meadows, at the level of individual flowers for all species except Asteraceae, which were sampled at the level of the capitulum. To identify an appropriate sampling scheme for meadow-wide resources, we conducted pilot surveys of A2 meadows in their first year at three Edinburgh sites in 2012 (Inch, Cairntows, Davidson’s Mains). We sampled between 49 and 99 quadrats for each meadow, and then used computer simulations (sampling actual transect values at random, without replacement) to assess the impact of sampling increasing numbers of 1m^2^ quadrats on the cumulative mean of nectar sugar mass and pollen volume per meadow (S1 and S2 Figs). In each meadow, variance in mean estimates declined sharply with increasing sample size, stabilising for each meadow and resource at a sample size of *ca*. 20 quadrats. We therefore sampled 20 x 1m^2^ quadrats per treatment replicate per sampling interval for the Edinburgh meadows during the main 2013 survey season (a total of 1400 quadrats over all treatments and sampling intervals). The 20 quadrats were sampled at 4 m intervals along the length of the plot, alternating between edge quadrats (i.e. extending from the plot edge to 1m into the plot) and internal quadrats (i.e. extending from 1m to 2m into the plot). For the three other cities, time constraints restricted sampling to a subset of the 20-quadrat scheme, comprising seven edge quadrats per treatment replicate per sampling interval located at 8m intervals along the plot length. We compare the consequences of adopting the seven or 20-quadrat scheme below using the Edinburgh data. In short, we consider the seven-quadrat estimates adequate for comparison of mean differences between treatments, and all comparisons among cities are based on data for the seven-quadrat scheme. Total numbers of flowers (or capitula) per treatment replicate for each species were estimated as the product of mean floral density/m^2^ x meadow area (m^2^). In 2013, over the four cities, 80 plots and six sampling intervals, we recorded over two million flowers of 105 plant species, from a total of 3068 quadrats.

To illustrate variation among replicates we present detailed data summaries for each of the Edinburgh treatment replicates, and present the data for each meadow location in the other three cities in S4 and S5 Figs. To allow comparison between cities, treatments and sampling time points, we use means calculated for each treatment across all time points, replicates and cities, as appropriate. We excluded from these values data for four sites at which management issues (detailed below) led to complete failure of the treatment relative to others in the same group. These comprise two perennial sites and two A1 annual sites (S2 Table).

Resources per meadow (nectar sugar mass or pollen volume) were calculated as the sum across plant species of (resource per flower) x (the number of flowers in the treatment replicate). 95% confidence intervals for resources are based on error estimates in the floral counts.

### Statistical methods

Statistical analyses were carried out in R version 3.1.3 (R Foundation for Statistical Computing, Vienna, Austria. 2015). To visualise seasonal trends in resource availability, means and 95% confidence intervals were plotted with the {ggplot2} package [63]. Correlations between each of nectar sugar and pollen volume and flower counts per transect were plotted with {PerformanceAnalytics} [64], and ranked dotplots were plotted with {stats} [65]. Flower counts (per species and summed across species) and resource values showed non-normality and heteroscedasticity that could not be corrected using standard data transformations. Variation of floral resource estimates among treatments was therefore tested using global Kruskal-Wallis tests on means calculated across all surveys for a given type (i.e. n = 7 quadrats x 5 replicates/city x 4 cities x 6 survey rounds) in {PMCMR} [66]. Pairwise comparisons between treatment means were tested using the post-hoc Tukey-Kramer-Nemenyi test with Chi-square correction for ties in {PMCMR}.

Variation in the composition of planted floral meadows within a meadow treatment between cities and sampling points was tested using the manyglm function of the R package {mvabund} [67]. This approach allows identification of multivariate patterns and fitting of a separate Generalised Linear Model (GLM) for each flowering species using a common set of explanatory variables. Resampling-based hypothesis testing within mvabund was then used to make community-level and taxon-specific inferences about which factors were associated with the multivariate abundance patterns. For these analyses we used mean floral counts/m^2^ from seven quadrats for each treatment replicate (Annual A1, Annual A2, Perennial) per survey round (May, June, July, early August, late August, September) per city (Bristol, Edinburgh, Leeds, Reading). For this dataset we specified a negative binomial error distribution, and checked assumptions of mean-variance and log-linearity as detailed in [67], both by plotting directly and by plotting residuals versus fitted values. We used Monte Carlo bootstrapping to estimate p-values adjusted to control the family-wise error rate across species, at the default setting of 1000 resamples. Variation in floral composition was visualised using non-metric multidimensional scaling (NMDS) with the metaMDS function of {vegan} [68], using a Bray-Curtis distance matrix calculated using mean floral counts (capitula for Asteraceae) per species per m^2^ for each meadow location and sampling time point. Meadows with fewer than 10 flowers (or capitula) in total per m^2^ were excluded from the analysis. Because NMDS only uses rank information and maps ranks non-linearly onto ordination space, it can handle non-linear species responses of any shape and robustly find underlying gradients. As the final ordination is partly dependent on the initial configuration, we used up to 99 consecutive NMDS iterations with random starting configurations to test for stability of the result.

All raw data, metadata and R code are available from the authors on request.

## Results

### Resource per flower

#### (a) Nectar sugar

**Fig 1:**
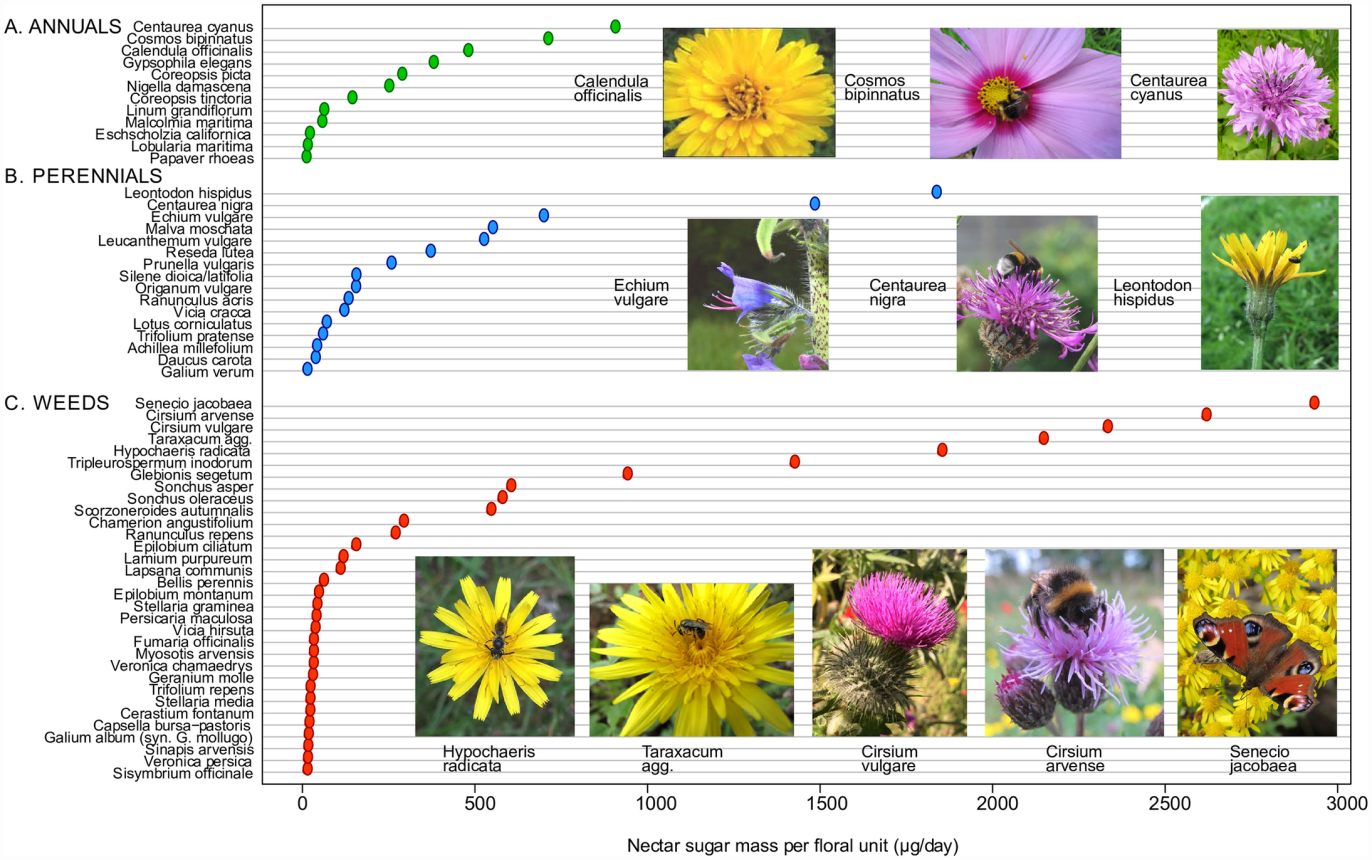
Mean nectar sugar mass per 24h per floral unit for species in A. the annual seed mix, B. the perennial seed mix, and B. native weeds in either mix. Values shown are ranked means in each group (mean values and standard errors are provided in S1 Table). Images of the top ranked species in each group are shown, with the highest-ranked at right. Images are provided by the project team with the exceptions of *Echium vulgare* (author: Ewan Cole) provided in 2016 under a (CCAL) CC BY 4.0 license from the Urban Flora of Scotland project.

Nectar sugar estimates per 24h for the seed mix species and associated weeds are shown in Fig 1 and S1 Table. Most of the highest values were for Asteraceae, for which a floral unit comprises an inflorescence (capitulum) rather than a single flower (Fig 1). The top-ranked annual species/floral unit/24h were *Centaurea cyanus* (896 ± 174μg s.e.m.), *Cosmos bipinnatus* (701 ± 69μg) and *Calendula officinalis* (470 ± 11μg). The top-ranked perennial species were *Leontodon hispidus* (1827 ± 193μg), *Centaurea nigra* (1474 ± 76μg) and *Echium vulgare* (688 ± 103μg). The top-ranked weed species, all native Asteraceae, produced more nectar sugar per floral unit than any seed mix species: *Senecio jacobaea* (2921 ± 448μg), *Cirsium arvense* (2609 +/- 239μg), *Cirsium vulgare* (2323 ± 418μg), and *Taraxacum* agg. (2137 ± 286μg). Both the annual and perennial mixes contained species with very low nectar sugar rewards per floral unit, including species with large individual flowers such as *Papaver rhoeas* (0.6 ± 0.6μg) and *Eschscholzia californica* (10 ± 1μg) in the annual mix, and species sampled as small individual flowers such as *Lobularia maritima* (4 ± 1μg) in the annual mix and *Daucus carota* (27 ± 7μg) and *Galium verum* (3.2 ± 0.6μg) in the perennial mix.

#### (b) Pollen

Total pollen rewards per floral unit were highest in Asteraceae sampled at the level of the entire capitulum (*Leucanthemum vulgare*, 15.9 ± 2μl, *Cosmos bipinnatus* 13.8 ± 1.9μl), and lowest in those species sampled at the level of small individual flowers (e.g. *Myosotis arvensis*, 0.0004 ± 0.0001μl) (S1 Table). Of species sampled at the level of a single flower, by far the most rewarding were the poppies, *Papaver rhoeas* (13.3 ± 2.8μl) and *Eschscholzia californica* (8.3±1.1μl). Among weed species, values were again highest for Asteraceae, including *Glebionis segetum* (5.1 ± 0.9μl) (presumed to be a garden escape) and native *Taraxacum* agg. (2.8 ± 0.7μl).

**Fig 2:**
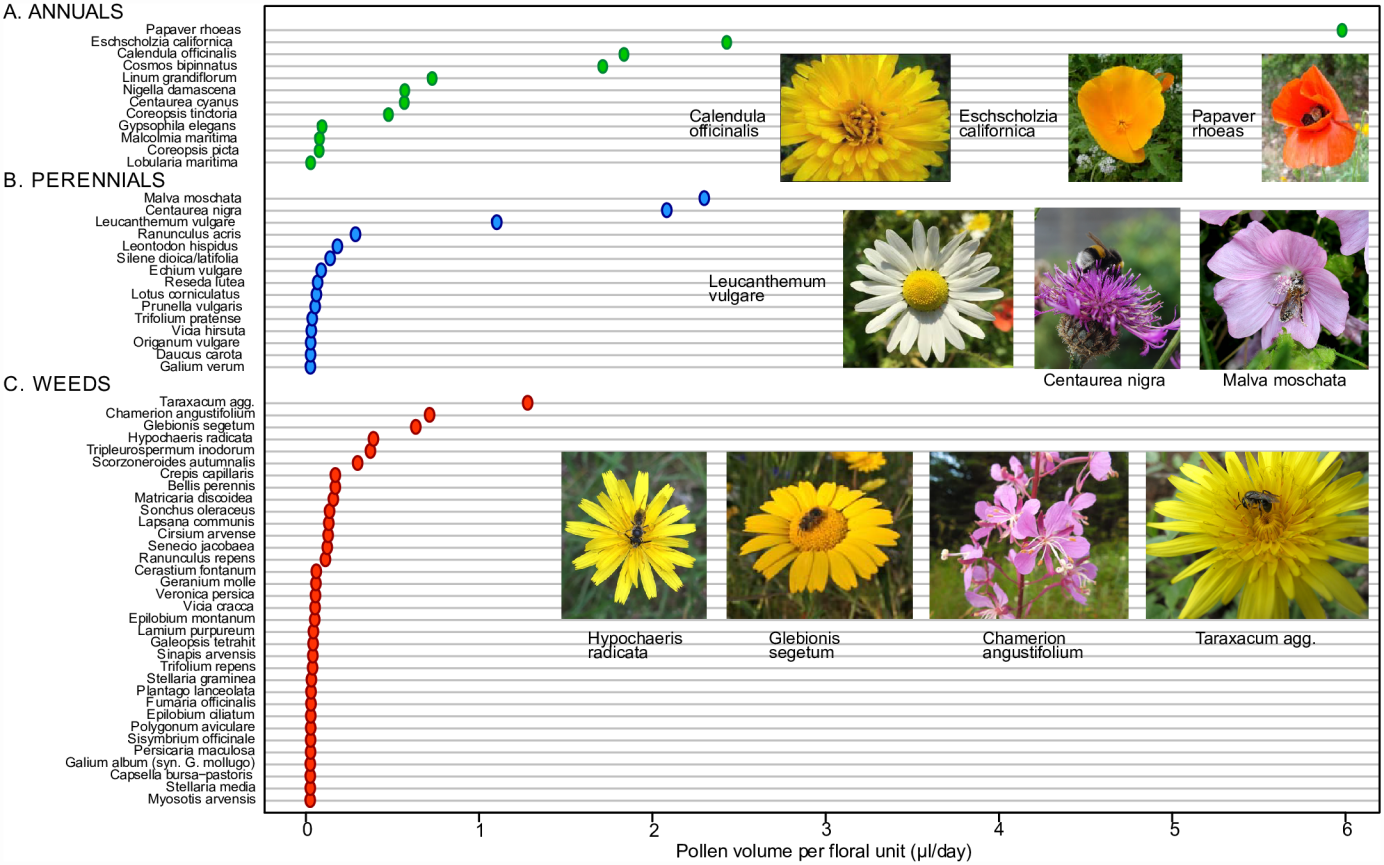
Mean pollen volume per 24h per floral unit for species in A. the annual seed mix, B. the perennial seed mix, and C. native weeds in either mix. Values shown are ranked means in each group. Mean values and standard errors, longevity and pollen volume/floral unit are provided in S1 Table). Images of the top ranked species in each group are shown, with the highest-ranked at right. Images are provided by the project team with the exceptions of *Chamerion angustifolium* (author: Ewan Cole) provided in 2016 under a (CCAL) CC BY 4.0 license by the Urban Flora of Scotland project.

Quantifying the contribution of each species to daily pollen resource provision at the meadow level requires scaling of total pollen volume by floral longevity, which ranged from mean values of a single day (e.g. *Cerastium fontanum*, *Veronica persica*, *Vicia hirsuta*) to 14.8 days (*Leucanthemum vulgare*), and was generally higher in Asteraceae species sampled at the level of the capitulum (see Fig 2 and S1 Table). The highest pollen rewards/floral unit/24h were provided by annual mix species; flowers of *Papaver rhoeas* (6.0μl pollen) provided more than twice the values for the next-ranked species, *Eschscholzia californica* (2.4μl) and *Calendula officinalis* (1.8μl; this species ranked highly for both nectar and pollen rewards). In the annual mix, *Coreopsis picta* (0.7μl) had a very low pollen reward per capitulum in comparison to other Asteraceae sampled. The top-ranked perennial mix species by floral unit were *Malva moschata* (2.3μl), *Centaurea nigra* (2.1μl; this species ranked highly for both nectar and pollen rewards) and *Leucanthemum vulgare* (1.1μl). The top-ranked weed species in our meadows were native *Taraxacum* agg. (1.25μl) and *Chamerion angustifolium* (0.7μl).

#### (c) Statistical prediction of resource per flower from floral morphology

Laboratory estimates of total pollen volume per floral unit were predicted by a linear model using anther size and stamen number as predictors (S3 Fig; adjusted R^2^ = 0.7025, F_4,54_ = 35.23, p<0.0001). The best-fit model was:

$$Pollen\;volume\;per\;single\;flower\;\left(\mu l\right)=\;e^{\left(2.34\;-8.98a-3.28b-5.59c-4.49d\right)}$$

where a = 1 / stamen-number-per-flower and b-d are binary scores (i.e. with values of 0 or 1) indicating the anther size class for the species (S3 Table) as medium (b), small (c) or tiny (d).

For half of the 59 species in our modelling dataset (S3 Table), fitted values were within the confidence limits of laboratory estimates. The model tends to overestimate values for flowers containing very low pollen volumes (S3 Table) although the magnitude of the error here is invariably in hundredths of a μl or less. Application of the model to the Agriland validation dataset found predicted values to (i) be highly correlated with measured values (n = 43, adjusted R^2^ = 0.76, p<0.001), and (ii) fall within the cloud of values observed in this study (S3 Fig). This suggests that the statistical model could be used to estimate values where no direct measurement is possible or available, and to identify outlying measured values that should perhaps be confirmed.

### Variation in meadow floral composition

**Fig 3:**
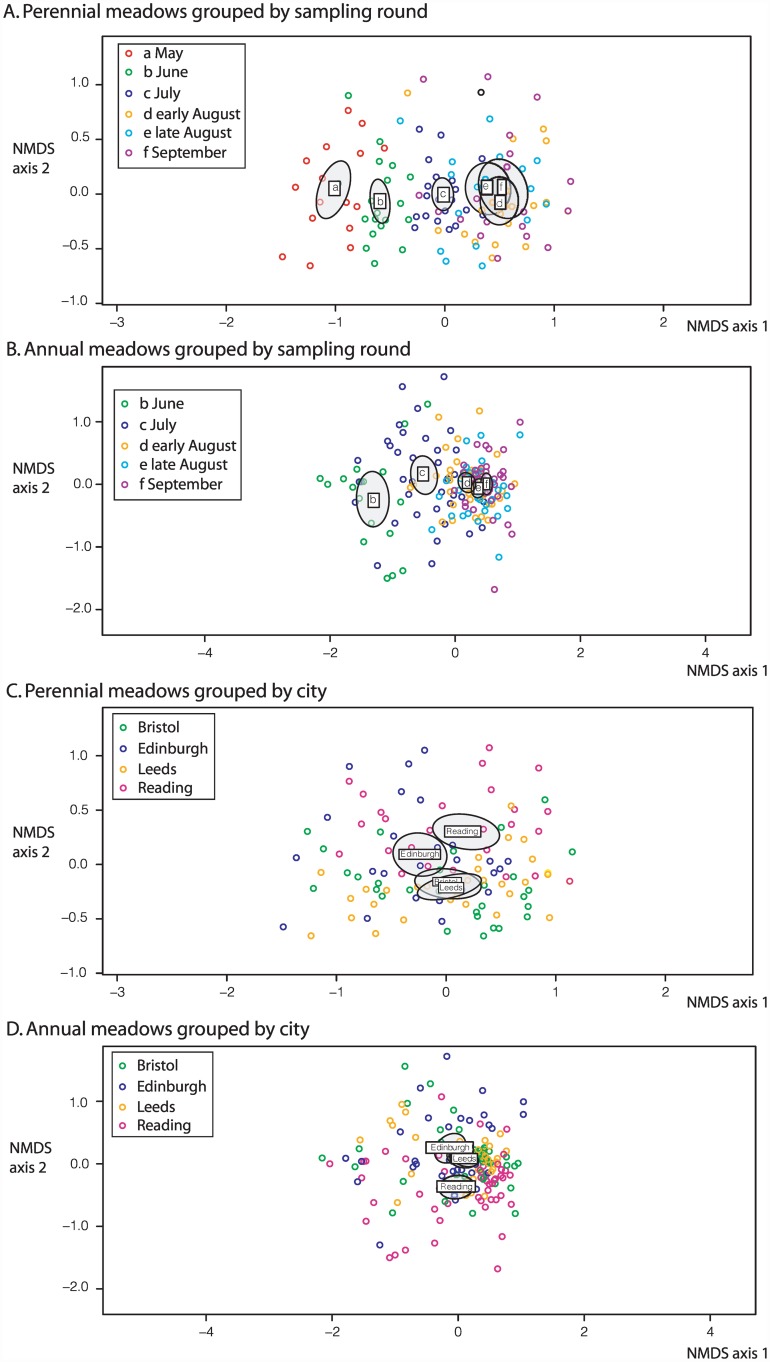
Non-metric multidimensional scaling (NMDS) analyses of changes in the composition of floral meadows through time and among cities. A. Perennial meadows across all cities, separated by survey round. B. Annual meadows across all cities, separated by survey round. In A and B, letters and ellipses show the centroid and 95% confidence limits for each survey round. C. Perennial meadows across all survey rounds, separated by city. D. Annual meadows across all survey rounds, separated by city. In C and D, letters and ellipses show the centroid and 95% confidence limits for each city. In D. the centroid for Bristol is hidden by, and almost identical to, the centroid for Leeds.

The floral composition resulting from each seed mix treatment varied through the season and among cities (Fig 3) (negative binomial manyglm, p<0.05 in each case). The two annual treatments, A1 and A2, also differed in city-specific ways (significant city x treatment interaction, p<0.05). Examination of the single species analyses (S4A Table) shows that for perennial meadows, among-city differences were primarily due to varying floral abundance of three of the sown species (in declining rank of deviance explained, *Achillea millefolium* 4.7%, *Malva moschata* 3%, *Reseda lutea* 2.8%, all p<0.001) and a suite of native weed species, including *Stellaria media*, *Crepis capillaris*, *Epilobium spp*., *Capsella bursa-pastoris*, *Polygonum aviculare* (all p<0.01), and *Taraxacum* agg. (p<0.03). The seed mix species *Achillea millefolium* and *Echium vulgare* and the weed species *Taraxacum* agg. achieved higher floral unit densities in the north (Edinburgh, Leeds) than in the south (Reading and Bristol), while *Knautia arvensis* showed the opposite trend.

For annual meadows (S4B Table), among-city differences were also due to varying floral abundance of a range of sown species (*Lobularia maritima*, *Malcolmia maritima* and *Cosmos bipinnatus*, all p<0.001), and native weed species including *Polygonum aviculare*, *Scorzoneroides autumnalis*, *Achillea millefolium*, *Crepis capillaris*, *Epilobium* spp., *Veronica* spp. and *Sonchus oleraceus* (all p<0.001). The mix species *Centaurea cyanus* achieved higher floral unit densities in the north (Edinburgh, Leeds) than in the south (Reading and Bristol), while *Malcolmia maritima* and the non-mix species *Sonchus oleraceus* and *Coreopsis* spp. showed the opposite trend. Differences between the A2 and A1 annual treatments (S4C Table) were due to significantly greater weed intrusion in A2 meadows, particularly *Veronica* spp. (7.9% deviance, p<0.05). The significant treatment x city interaction for annual meadows was predominantly due to variable performance of the sown species *Calendula officinalis*, which had higher floral density in A1 than A2 meadows in Edinburgh and Leeds, while showing the opposite pattern in Bristol and Reading.

A general pattern across mixes and replicates is that flower counts were commonly dominated by high values for a small number of species, with a distribution tail of species with very low counts. For example, the highest number of flowers recorded for any single m^2^ in a perennial meadow was 34 143 (Edinburgh, Pilrig Park perennial meadow on 30 July 2013), of which 97.8% were contributed by *Daucus carota*, 2.1% by *Achillea millefolium*, and the remainder by three much rarer species. The highest values recorded for a single m^2^ in an annual meadow was 23 704 (Bristol, Hengrove Farm on 9 August 2013), of which 99.1% were contributed by *Lobularia maritima*, with the remainder contributed by four very rare species. The same superabundant species showed the highest mean densities across all transects, cities and time points (*Daucus carota*, 1762 flowers/m^2^ and *Lobularia maritima* 580 flowers/m^2^). Mean flower densities for the rarest seed mix species were 3–4 orders of magnitude lower (perennials *Stachys sylvatica* 0.034 flowers/m^2^ and *Knautia arvensis* 0.095 flowers/m^2^, annual *Nigella damascena* 0.088 flowers/m^2^). Many of the weed species recorded in our surveys had a mean density over all sampled quadrats of less than 0.1 flowers/m^2^ (*Aethusa cynapium*, *Helminthotheca echioides*, *Geranium dissectum*, *Veronica serpyllifolia*, *Agrimonia eupatoria*, *Fallopia convolvulus*, *Epilobium tetragonum*, *Crepis vesicaria*, *Alliaria petiolata*, *Cirsium vulgare*, *Glebionis segetum*, *Stachys sylvatica*, *Viola* spp., *Potentilla reptans*, *Spergula arvensis*, *Leontodon saxatilis*, *Arabidopsis thaliana*, *Veronica persica*, *Calystegia sepium*, *Chamaemelum nobile*, *Papaver somniferum*, *Ranunculus bulbosus*).

Variation in meadow composition between cities and time points as revealed by NMDS and the two dominant axes of floral variation is shown in Fig 3. Stress values for these analyses were 0.216 for annual meadows and 0.200 for perennial meadows, indicating that much variation in the data remains unexplained. Repeated iterations of NMDS nevertheless produce very similar patterns in each dataset. The plots of samples within treatment separated by sampling round (Fig 3A and 3B) show a significant ordering by phenology (given non-overlapping 95% confidence intervals around the centroid for each sampling round), moving from left to right in ordination space through the year (i.e. b = June, c = July, d = early August, e = late August, f = September) corresponding with the first NMDS axis.

The same data for each treatment partitioned by city (Fig 3C and 3D) present an alignment with the second NMDS axis. No clear divergence among cities is apparent, although for annual meadows Bristol and Leeds group more closely with each other than with Edinburgh. Regional drought aside, the implication is that for each seed mix treatment, variation in meadow composition and abundance depends strongly on season and less on geographical position in the UK.

### Floral resources at the meadow level

#### (a) Nectar sugar

Across all four cities and time points, mean nectar sugar production/day/m^2^ varied significantly among treatments (global Kruskal-Wallis test, 3 d.f., Chi-square = 128.93, p<0.01). All planted meadow treatments produced significantly more nectar sugar than amenity grassland controls (Table 1). Perennial meadows produced significantly more nectar than both A1 and A2 annual meadow treatments, which did not differ significantly from one another (Table 1).

**Table 1:** Mean, median and quartile values for nectar sugar mass/m^2^/day for each meadow treatment type across all cities and surveys, showing significant difference groups (p < 0.05) by the post-hoc Tukey-Kramer-Nemenyi test.

Meadow treatment	Mean nectar sugar mass (mg)	Median nectar sugar mass (mg)	25^th^ percentile	75^th^ percentile	Significance group
Perennial	67.547	35.981	6.076	83.603	a
Annual A1	10.821	3.072	0.013	17.015	b
Annual A2	10.594	5.129	0.148	13.277	b
Amenity grassland Control	0.486	0.090	0.000	0.367	c

**Fig 4:**
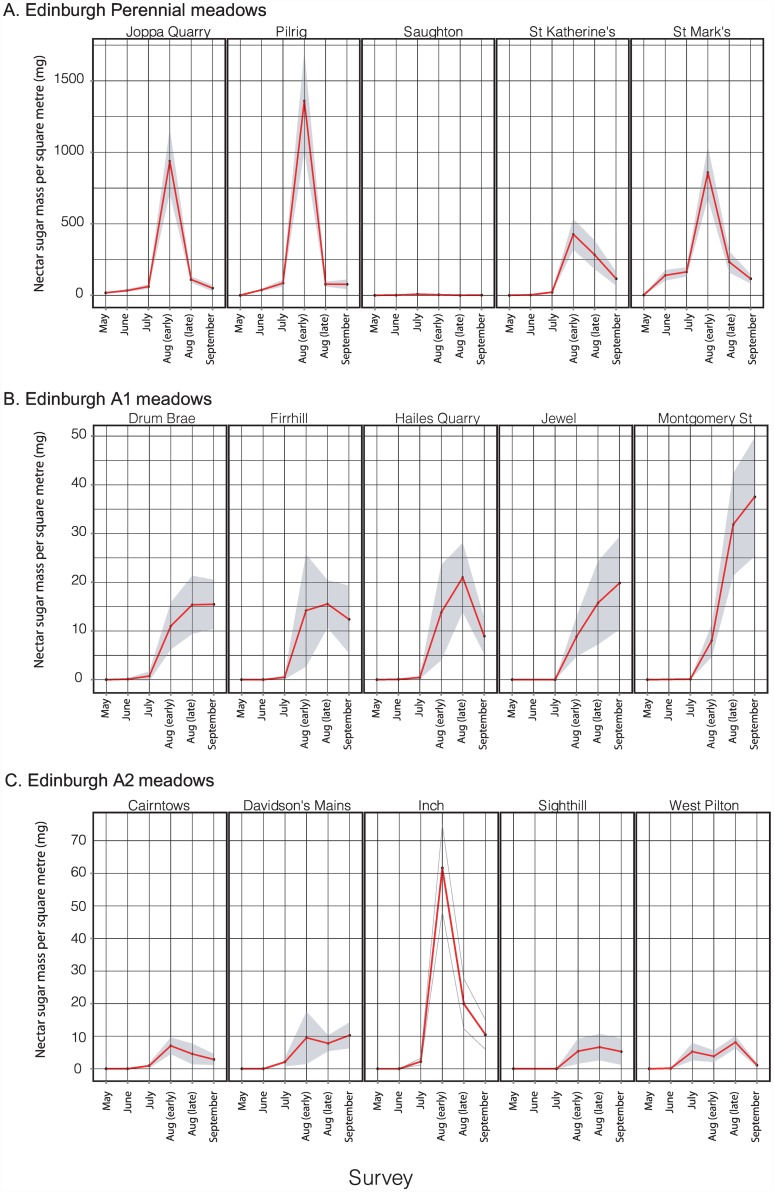
Seasonal patterns in daily nectar sugar availability for individual Edinburgh meadows sown with A. perennial, B. annual A1, and C. annual A2 treatments. Data were sampled at three-week intervals through 2013. Values at each time point are means based on 20x 1m^2^ quadrats, with dark shading showing 95% confidence limits. The poor performance of the Saughton perennial meadow was due to accidental mowing, and this replicate was excluded from statistical analyses.

Changes in meadow-level nectar sugar mass through the 2013 season are shown for the five Edinburgh replicates in each of the A1, A2 and P treatments in Fig 4; equivalent plots for the other three cities are shown in S4 Fig. The Edinburgh meadows show the following patterns (Fig 4): (i) Productivity per square metre was higher in the perennial compared to annual meadows, with a mean exceeding 1g/day/m^2^ at Pilrig. One perennial meadow (Saughton, excluded from statistical analyses) performed very poorly due to accidental mowing. Peak values for both annual treatments were one to two orders of magnitude lower at 10–60mg/day/m^2^. Nectar sugar values for amenity grassland control sites were much lower than in all planted meadow treatments: for 29 of the total of 30 control site surveys (five Edinburgh control sites x six sampling points), mean nectar sugar/day/m^2^ was less than 1mg. The maximum value for any control site was for the late June survey at Morningside Park, with a mean value of 11.3 mg/day/m^2^, which had not been recently mown. (ii) Productivity/day/m^2^ varied substantially through the season for all treatments. Nectar productivity of perennial meadows peaked earlier in the year (early August for all replicates) than for annual A1 meadows (which all peaked in late August or September). Annual meadows produced little or no nectar in June and July. (iii) The temporal pattern was most variable for A2 meadows. (iv) Nectar productivity was most consistent among annual A1 replicates.

**Fig 5:**
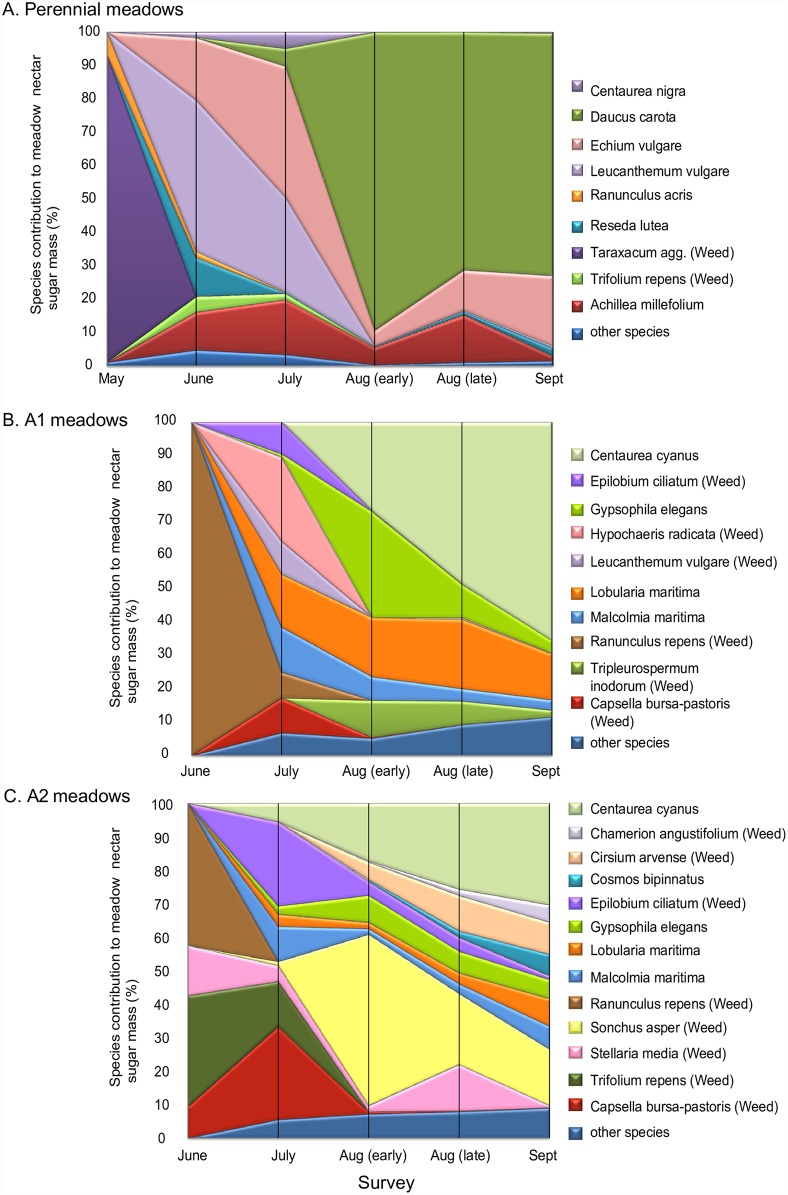
Seasonal patterns in the proportion of available daily nectar sugar contributed by individual plant species for Edinburgh meadows sown with A. perennial, B. annual A1, and C. annual A2 treatments. The percentage of total meadow nectar sugar mass attributable to each species is indicated by the height of the filled polygon for that species at a given seasonal time point. Values at each time point are based on 100x 1m^2^ quadrats across 5 replicate meadows at each time point for each meadow treatment. Note that in both annual and perennial treatments, native perennial weeds provided up to 100% of nectar and pollen resources early in the year.

The contribution by individual species to nectar sugar in each Edinburgh meadow treatment is shown in Fig 5. Most of the nectar sugar was provided by one or a few species at a given seasonal time point. A high proportion of early season nectar production in all treatments was contributed by native weed species—particularly *Taraxacum* agg.—in the perennial meadows (providing almost all of the limited nectar supplies for this treatment in May), *Ranunculus repens* in the A1 meadows (providing almost all of the limited nectar supplies for this treatment in June), and *Ranunculus repens* and *Trifolium repens* in the A2 meadows. The high midsummer peak in nectar production at Edinburgh’s Inch Park A2 replicate was largely due to high abundance of the seed mix species *Centaurea cyanus* and the native weed *Sonchus asper*. The high early August peak in nectar production of perennial meadows was largely due to abundant flowering of *Daucus carota*, and to a lesser extent *Achillea millefolium* and *Echium vulgare*.

**Fig 6:**
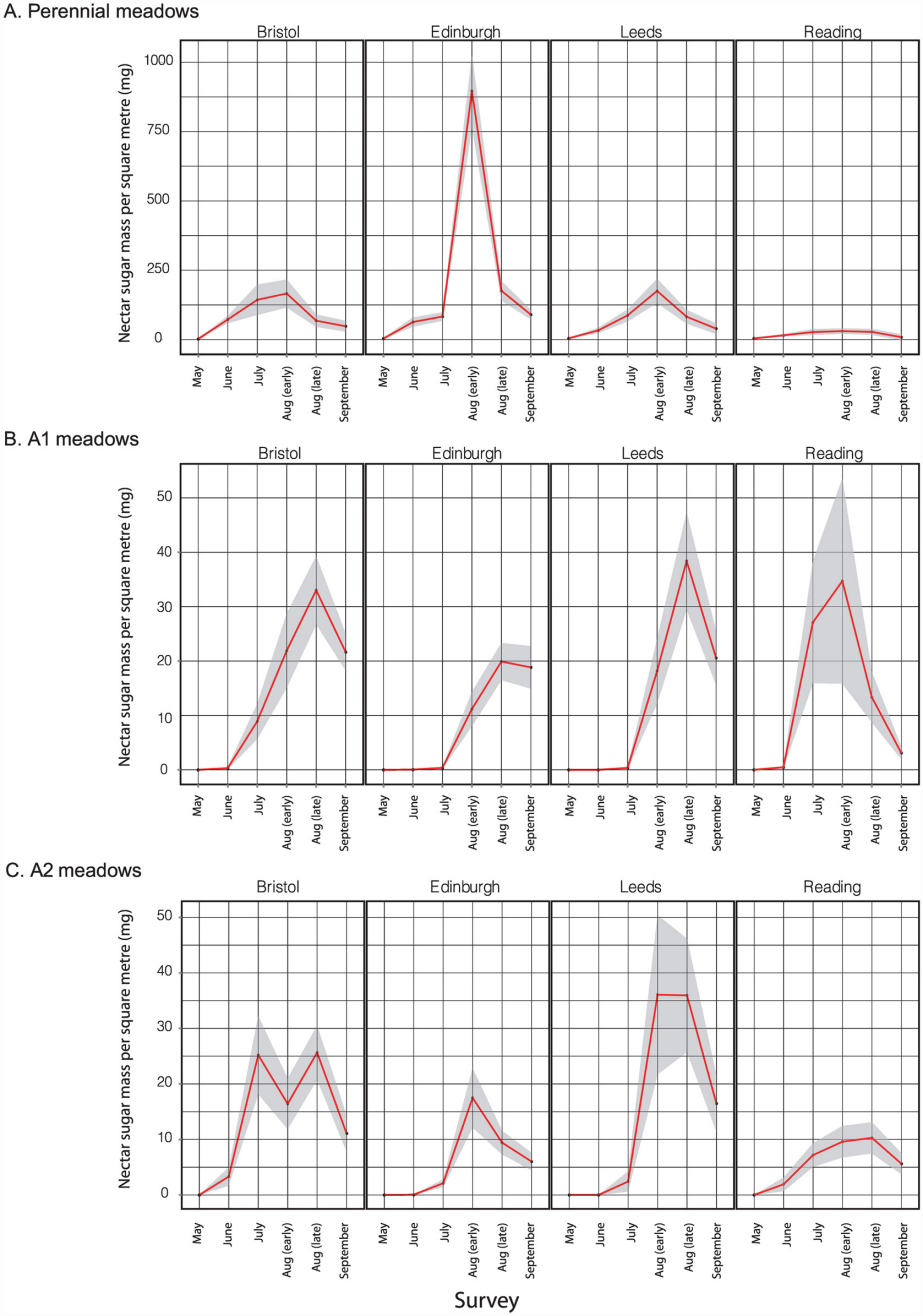
Seasonal patterns in nectar sugar availability across four UK cities for meadows sown with A. perennial, B. annual A1, and C. annual A2 treatments, assessed at three-week intervals in 2013. Values at each time point are means based on 7x 1m^2^ quadrats, with dark shading showing 95% confidence limits. Plots for individual sites are shown for Bristol, Reading and Leeds in S3 Fig.

Consideration of the other three cities (Fig 6 and S4 Fig) shows that perennial meadows again had higher nectar abundance than both A1 and A2 annual treatments in Bristol and Leeds, and that nectar productivity in perennial meadows again peaked earlier in the year than in A1 meadows. Perennial meadows in Reading performed poorly relative to other cities, and showed no clear seasonal peak in nectar production (see Discussion). There is some evidence of a latitudinal effect in the annual meadow treatments, with nectar production increasing earlier in the year in southern cities (June and July in Bristol and Reading) than further north (early August in Edinburgh and Leeds). Whilst per-species estimates using the seven-quadrat sampling regime are subject to the stochastic patterns shown in S1 and S2 Figs, the same species that dominated perennial nectar sugar production in Edinburgh also dominated in the other three cities: over all cities, replicates and time points, *Daucus carota* contributed 56.5% of all nectar sugar. The annual meadows showed more heterogeneous patterns of species abundance and resource contribution, with *Centaurea cyanus* making the greatest contribution overall to nectar (33.19%). Each mix contained some species that contributed very little nectar sugar. The two lowest mean sugar mass/day/m^2^ values across all cities, replicates and time points for their treatments were the perennial *Galium verum* (mean 22.69μg/day/m^2^, 0.03% of total daily nectar sugar) and the annual *Papaver rhoeas* (mean 1.63μg/day/m^2^, 0.008% of total daily nectar sugar). Across all cities, replicates and time points, seven species in the perennial mix (*Galium verum* 0.027%, *Trifolium pratense* 0.083%, *Malva moschata* 0.129%, *Ranunculus acris* 0.329%, *Lotus corniculatus* 0.359%, *Vicia cracca* 0.383%, and *Origanum vulgare* 0.581%) and three in the A1 annual mix treatment (*Papaver rhoeas* 0.0083%, *Nigella damascena* 0.23% and *Eschscholzia californica* 0.25%) contributed less than 1% of total nectar production.

#### (b) Pollen

Across all four cities, pollen production varied significantly among treatments (global Kruskal-Wallis test, 3 d.f., Chi-squared = 183.55, p<0.01). All planted meadows produced significantly more pollen than amenity grassland controls (Table 2). Across the season, perennial meadows produced significantly more pollen than annual A1 meadows, while A1 and A2 annual treatments produced did not differ significantly (Table 2).

**Table 2:** Mean, median and quartile values for pollen volume /m^2^/day) for each meadow treatment type across all cities and surveys, showing significant difference groups (p < 0.05) by the post-hoc Tukey-Kramer-Nemenyi test.

Meadow treatment	Mean nectar sugar mass (mg)	Median nectar sugar mass (mg)	25^th^ percentile	75^th^ percentile	Significance group
Perennial	0.0549	0.012	0.004	0.045	a
Annual A1	0.0327	0.005	0.000	0.041	b
Annual A2	0.0287	0.013	0.000	0.048	ab
Amenity grassland Control	0.0005	0.000	0.000	0.001	c

**Fig 7:**
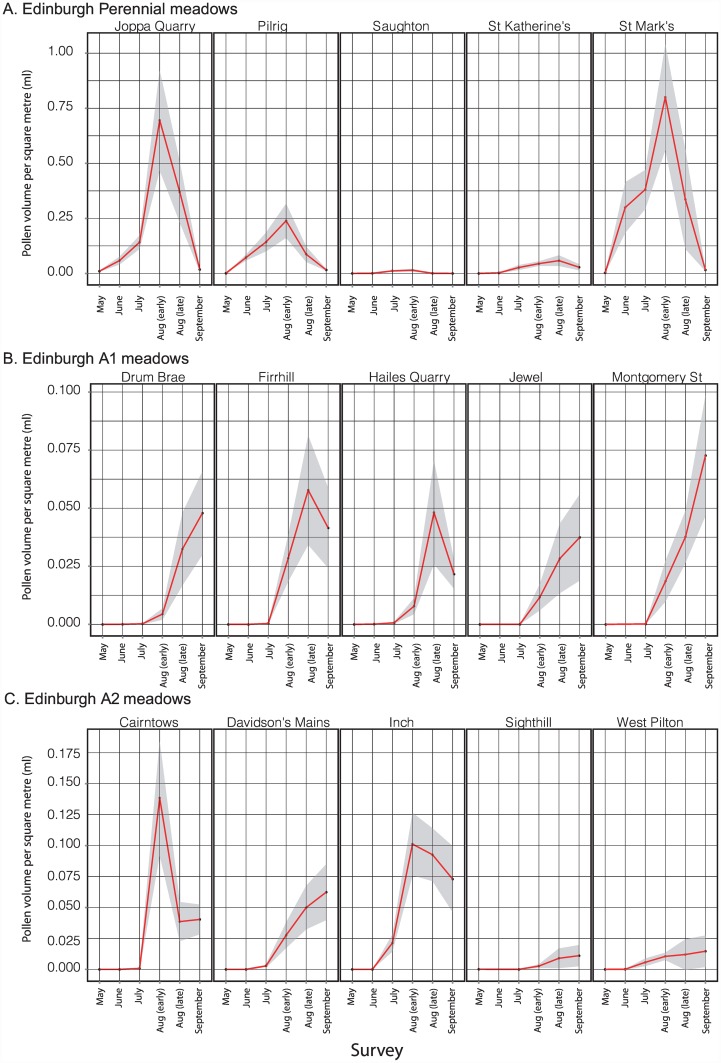
Seasonal patterns in daily pollen availability for individual Edinburgh meadows sown with A. perennial, B. annual A1, and C. annual A2 treatments. Data were sampled at three-week intervals through 2013. Values each time point are means based on 20x 1m^2^ quadrats, with dark shading showing 95% confidence limits.

Changes in meadow-level pollen volume through the 2013 season are shown for the five Edinburgh replicates in each of the A1, A2 and perennial treatments in Fig 7; equivalent plots for the other three cities are shown in S5 Fig. The Edinburgh meadows show the following patterns (Fig 7): (i) Productivity per square metre was highest in the perennial meadows, with a mean of approx. 0.75ml/day/m^2^ at Joppa Quarry and St. Marks. Peak values for both annual treatments were an order of magnitude lower at 0.01–0.07ml/day/m^2^. Peak values for amenity grassland control sites were usually two to three orders of magnitude lower than for any planted sites: for 29 of the 30 Edinburgh control site surveys (five sites x six sampling points) through the season, mean pollen volumes were less than 0.0001ml/day/m^2^. The highest value for any control site of 0.0058 ml/day/m^2^ was recorded in June for Morningside Park, which also showed the highest nectar sugar resource of any control site. (ii) Productivity/m^2^ varied substantially through the season for all treatments. Pollen productivity of perennial meadows peaked earlier in the year (early or late August) than for annual A1 meadows (late August or September). Annual meadows produced little or no pollen in June and July (due to low floral abundance, rather than presence of species offering only low rewards/flower). (iii) Temporal patterns and productivity were most variable for A2 meadows. (iv) Pollen productivity was most consistent among annual A1 replicates; two perennial meadows (Saughton and St. Katherine’s) showed low resource levels due to management problems (accidental mowing and inadequate ground preparation prior to seeding, respectively). Low pollen production at two Edinburgh A2 meadows (Sighthill and West Pilton) was associated with high abundance of weeds, particularly *Rumex* spp. and *Chenopodium* spp. that offer low pollen volumes per flower.

**Fig 8:**
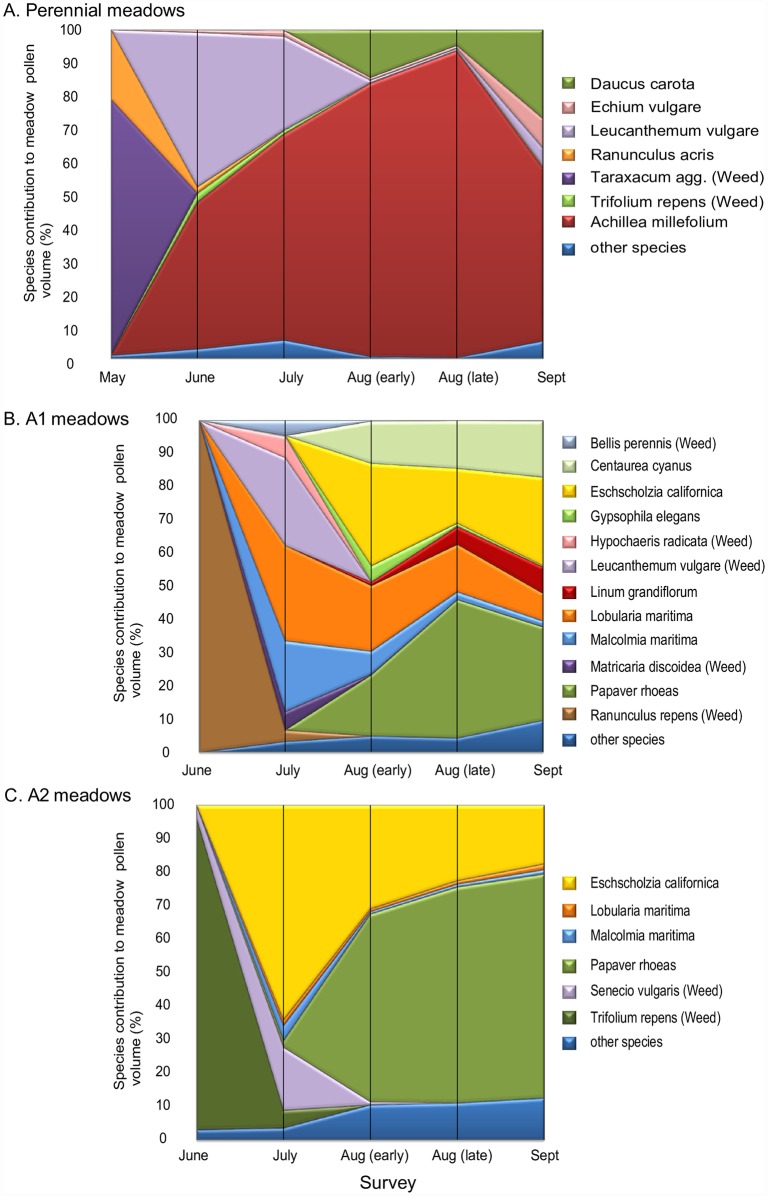
Seasonal patterns in the proportion of available daily pollen contributed by individual plant species for Edinburgh meadows sown with A. perennial, B. annual A1, and C. annual A2 treatments. The percentage of estimated total meadow pollen volume attributable to each species is indicated by the height of the filled polygon for that species at a given seasonal time point. Values at each time point are based on 100x 1m^2^ quadrats across 5 replicate meadows at each time point for each meadow treatment. Note that in both annual and perennial treatments, native perennial weeds contributed up to 100% of nectar and pollen resources early in the year.

The contribution by individual species to pollen volumes in Edinburgh meadow treatments is shown in Fig 8. As for nectar, most pollen was provided by a few species at a given seasonal time point, particularly in perennial and A2 meadows. Native weeds contributed almost all early-season pollen production in all treatments—particularly *Taraxacum* agg. in perennial meadows, *Ranunculus repens* in A1 meadows and *Trifolium repens* in A2 meadows. Later in the summer, perennial meadow pollen production was dominated by *Leucanthemum vulgare* and *Achillea millefolium*, while pollen production in the annual meadows (particularly A2) was dominated by the poppies *Papaver rhoeas* and *Eschscholzia californica*.

**Fig 9:**
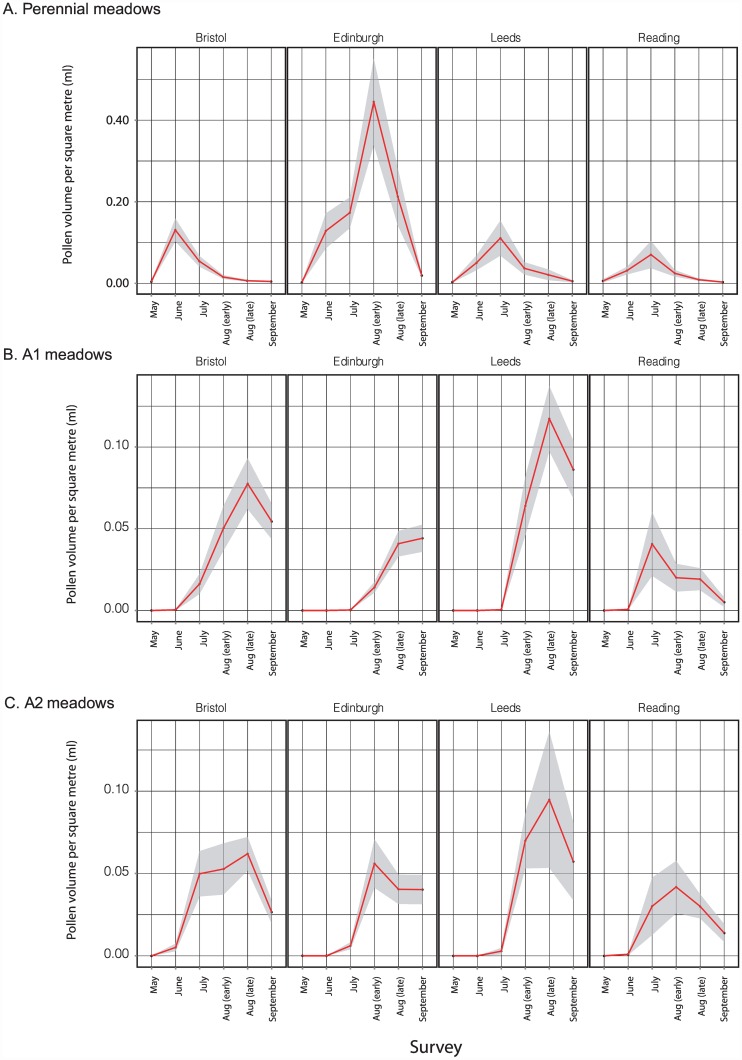
Seasonal patterns in pollen availability across four UK cities for meadows sown with A. perennial, B. annual A1, and C. annual A2 treatments, assessed at three-week intervals in 2013. Values at each time point are means based on 7x 1m^2^ quadrats, with dark shading showing 95% confidence limits.

Consideration of the other three cities (Fig 9 and S5 Fig) shows that, in contrast to Edinburgh, meadow pollen production was more comparable across perennial and annual meadow treatments. In Bristol and Leeds, productivity of perennial meadows again peaked before annual A1 meadows. There is some evidence of earlier A1 pollen production peaks in southern (Bristol and Reading) than in northern (Edinburgh and Leeds) cities, though this is less clear than for nectar. Whilst per-species estimates using the seven-quadrat sampling regime are subject to the stochastic patterns shown in S1 and S2 Figs, most of the same species that dominated pollen production in Edinburgh also dominated in the other three cities: over all cities, replicates and time points, dominant contributions to total perennial pollen volume were made by *Leucanthemum vulgare* (49.6%) and *Achillea millefolium* (37.9%), and to total A1 pollen volume by *Eschscholzia californica* (20.9%) and *Papaver rhoeas* (19.8%). However, Bristol did not show the high peak of *Achillea millefolium* seen in Edinburgh, highlighting between-site variation in the spectrum of species contributing pollen from a given seed mix. Both seed mixes contained species making very low contributions to pollen volume at the meadow level. Over all cities, replicates and time points, the perennial species producing the lowest mean pollen volume/day/m^2^ were *Galium verum* (0.067μl/day/m^2^, 0.014% of total pollen volume), *Origanum vulgare* (0.033μl/day/m^2^, 0.007%) and *Trifolium pratense* (0.029μl/day/m^2^, 0.006%), with a further seven species contributing less than 1% to total pollen volume (*Vicia cracca* 0.024%, *Prunella vulgaris* 0.034%, *Lotus corniculatus* 0.159%, *Reseda lutea* 0.168%, *Malva moschata* 0.219%, *Echium vulgare* 0.536% and *Ranunculus acris* 0.678%). Low values were due to low numbers of available flowers (e.g. *Origanum vulgare*, *Malva moschata*, *Echium vulgare*) and/or low resource levels per flower (e.g. *Galium verum*). In A1 treatments the lowest value was substantially higher, at 0.28μl/day/m^2^ (0.31%) for *Nigell adamascena*, with all other mix species contributing >1% to total pollen volume.

#### (c) Impact of transect sampling strategy on estimates of floral resources

Sampling of up to 99 x 1m^2^ quadrats in individual meadows followed by *in silico* resampling showed variance in estimates of floral abundance (and hence nectar and pollen resources) to decline with increasing numbers of quadrats. Comparison of the seven- and 20- quadrat sampling schemes for Edinburgh data shows that estimates for a given site and survey round could differ between schemes by up to 50%, but with no systematic direction to the difference. For example, at the peak of Edinburgh nectar sugar production in early August, the seven-quadrat estimate of daily nectar sugar mass/m^2^ was 16–17% higher than the 20-quadrat estimate for two perennial mix sites (Pilrig and St. Marks), but 24% lower for another (St. Katherine’s). For the Edinburgh perennial replicate that failed (Saughton), inclusion of 20 quadrats meant the difference between a mean value of zero (seven quadrats) and a very low value of 3.5mg/m^2^/day. Despite variation for individual sites, means over all five replicates for this treatment and time point were within 10% (718mg/m^2^/day for 20 quadrat estimates, total 100m^2^ sampled, and 778mg/m^2^/day for seven-quadrat estimates, total 35m^2^ sampled). Similar magnitudes of difference were observed for other Edinburgh treatments and time points. We thus consider the seven-quadrat estimates adequate for comparison of mean differences between treatments.

#### (d) The relationship between resource density and flower density

**Fig 10:**
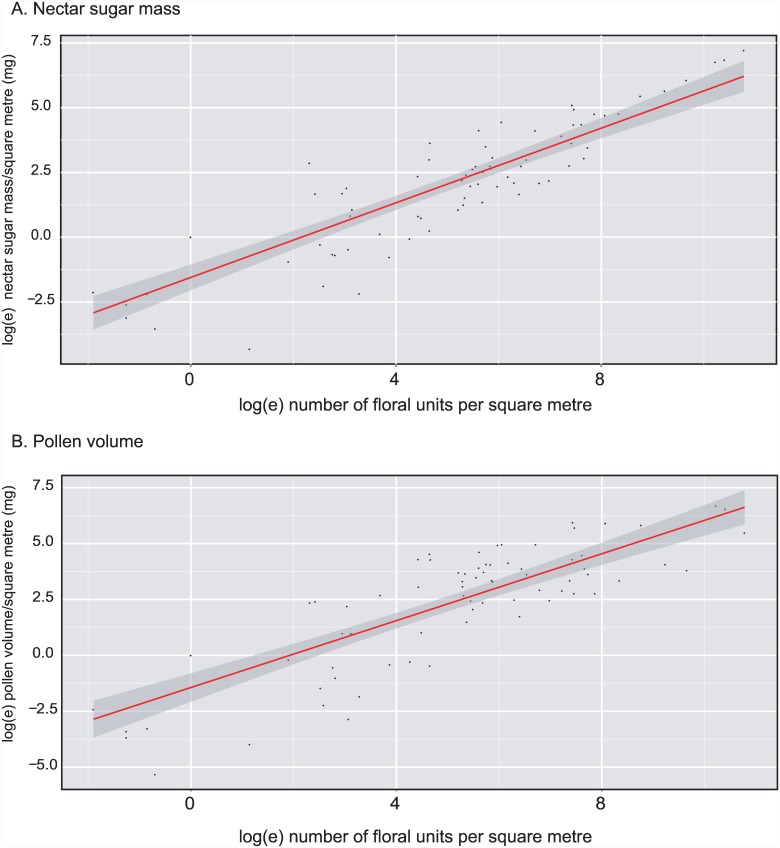
Relationships between counts of floral units and A. nectar sugar mass/m^2^, and B. pollen volume/m^2^ across all Edinburgh meadow surveys. Note that data have been log_e_ transformed. The fitted lines are least squares regressions of the transformed data, with the darker shaded area showing standard errors of fitted values. The fitted lines (± standard errors of estimates, n = 80 in each case) for A is y = 0.721(±0.045)x– 1.556(±0.251), and for (b) is y = 0.748(±0.057)x– 1.439(±0.320).

A plot of flower counts against floral resource (Fig 10) for all Edinburgh meadow surveys (n = 80) shows that both nectar sugar mass/m^2^ (adjusted R^2^ = 0.765, p<0.0001) and pollen volume/m^2^ (adjusted R^2^ = 0.683, p<0.0001) are highly correlated with flower count, with better predictive power for nectar sugar.

## Discussion

Our aims in this study were to quantify per-flower nectar sugar and pollen rewards for species in two seed mixes, and weeds from the seed bank that commonly grow with them in UK urban environments, and to estimate the total floral rewards these seed mixes provide per unit area when grown as urban meadows. We first discuss methodological issues related to our approach, before focussing on per species and per meadow resource estimates. Finally, we consider what more is needed to better estimate floral resource provision in urban and other habitats, and implications for urban green space managers.

### Methodological limitations in quantification of floral resources

Quantification of floral resources involves two key steps: estimation of nectar and pollen resources per flower per species and estimation of numbers of flower per species per unit area. Both processes have associated sampling error, and are subject to environmental and/or genetic variation within species.

(a) *Resources per flower*. The major challenge in estimating resources per flower lies in effective sampling of very small volumes of nectar or pollen in very small individual flowers or florets. Extraction of pollen through sonication is widely used and generally effective [69] and anthers can be visually checked to have released their pollen after sonication. In contrast it is much more difficult to be sure that very tiny volumes of nectar have been harvested: even when flowers of florets are flushed with a known volume of water, we cannot be sure that effective mixing of nectar and added water has occurred deep within the flower. We recorded zero values for nectar secretion using floret flushing in four species: *Crepis capillaris*, *Matricaria discoidea*, *Plantago lanceolata*, and *Senecio vulgaris*. Nectar has been collected from *Senecio vulgaris* by centrifuging individual capitula [70], and butterflies are known to feed (presumably on nectar) from *Matricaria discoidea* [71]. Previous studies have confirmed our finding of zero or very low nectar production in some species, including *Papaver rhoeas* (low but non-zero in [58], zero in [72]), *Eschscholzia californica* [73] and *Plantago lanceolata* [73]. Given the likelihood of failing to remove all resources from flowers, and possibility of false zeros for some taxa, we regard our floral resource estimates as underestimates. More detailed examination of nectar secretion in each flower type (e.g. following [74]) is clearly desirable.

Quantification of floral resources per unit area required estimation of flower densities for each species in each survey. Surveys of Edinburgh meadows using a range of sampling intensities showed sampling variance to decline with increased sampling effort, a pattern that reflects better sampling of spatial variation in sampled taxa among quadrats [75]. Nevertheless, comparison of results for seven and 20 quadrats across all of the Edinburgh surveys shows similar treatment-mean resource values with no systematic directional bias. Given that effects of flower sampling strategy and errors in estimation of resources per flower and floral longevity are compounded in estimation of floral resources per unit area, we see our approach as providing an order of magnitude estimate of meadow resource provision, rather than exact values.

An additional issue to address is the possibility of between-city variation in the resources provided by floral units of a given species drawn, as in our study, from the same mix of seed provenances. Variation in growth conditions (e.g. soil type or water availability) has been shown to cause variation in nectar and/or pollen resource levels per flower (e.g. [76,77]). In natural systems, such environmental variation is compounded by any population- or cultivar-associated genetic variation in floral resources (e.g. [77–79]). We illustrate the combined impact of these effects by comparing our resource values with nectar values generated for other populations of the same species in different locations, sampled using the same protocols by Baude *et al*. [57] (S7 Fig). Data for the two studies show broad agreement, with no apparent systematic bias, and two-thirds of the values are within each others’ 95% confidence intervals. Quantitative and qualitative (see below) variation in floral resource provision is an important area for future study.

### Cross-species variation in resources per flower, and consideration of resource quality

Both nectar and pollen resources per flower/capitulum varied enormously among the species in our surveys. In part this reflects differences in the scale of our sampling; it is unsurprising that we found the multi-floret capitula of Asteraceae to contain more resources than a single-flower floral unit of other taxa (all of the top 10 ranked floral units for nectar sugar mass and seven of the top ten for pollen volume were Asteraceae; Figs 1 and 2). Single flowers of *Papaver rhoeas* and *Eschscholzia californica* (Papaveraceae) offered the highest per unit pollen rewards, providing as much pollen per day as *ca*. 100 flowers of *Echium vulgare* and *Trifolium pratense*, or 1000 flowers of *Daucus carota* or *Origanum vulgare*. Our data confirm high levels of nectar (annual *Centaurea* spp.), pollen (*Eschscholzia californica*) or both (*Cosmos bipinnatus*) in several seed mix taxa whose value to bees has previously been questioned [80]. Relatively few species were highly rewarding for both pollen and nectar; the best joint performer was *Calendula officinalis*. It is striking that non-mix and native weed species contribute the top five ranked nectar producers per floral unit (including *Senecio jacobaea*, *Cirsium vulgare*, *C*. *arvense* and *Taraxacum* agg.) and two of the top ten-ranked pollen producers (*Taraxacum* agg. and *Chamerion angustifolium*). These results support previous studies showing the value of some weed species for bees and other taxa [81–83]. Even low numbers of floral units of any of the highly ranked seed mix or weed species would significantly increase floral resource quantity relative to amenity grassland.

There is growing evidence that resource quality, as well as quantity, matters for flower visitors. Pollens vary widely in their percentage protein by mass, and also in the amino acid composition of proteins present [53,84,85]. Flower visitors respond to this variation [54,84,86], and pollens rich in essential amino acids are associated with improved bee health [87] and population growth [88,89]. Protein percentage by mass, and the percentage of essential amino acids, are given for 23 of our study species in S1 Table. Though these data cover only a subset of species, they show that some mix species have more protein-rich pollens (*Echium vulgare* 44.1%, *Eschscholzia californica* 43.1%) than the weed species that contribute the highest per floral unit mass of nectar sugar (*Senecio jacobaea* 17.2%, *Cirsium arvense* 21.9%, *Cirsium vulgare* 22.1% protein, *Taraxacum* agg 19–22.7%) [90]. Furthermore, while the highest ranked seed mix species can have a high % by mass of essential amino acids (e.g. >39% for *Echium vulgare*), and are associated with high honey bee health (*Papaver rhoeas*, [91]), some of the weed species are known to be deficient. For example, *Taraxacum* spp. pollens lack the amino acids cysteine, tryptophan and phenylalanine and have low levels of valine, methionine, and isoleucine [52,53]. This is associated with demonstrated low nutritional value of *Taraxacum* pollen to some social bees [88,92]. *Ranunculus* pollen has also been identified as a low quality pollen source for honeybees [91]. These examples should not be taken to imply that weeds routinely have less nutritious pollen for bees than seed mix cultivars; pollen properties are strongly predicted by plant phylogenetic relationships, such that closely-related weed and seed mix species are predicted to have broadly similar pollens [53]. Data on a wider set of species would be extremely useful. It is also clear that flower visitors—and particularly solitary bees [93–95] vary in their attraction to, and ability to exploit, specific pollens—and specialist species can do well on plants that are less favoured by generalist bees [96]. Resource presentation in terms of floral functional groups and phenology (e.g. [97–99]) is also known to be an important determinant of resource availability to different groups of flower visitors. For example whilst *Eschscholzia californica*, *Malva moschata* and *Centaurea nigra* produce similarly high pollen volumes per floral unit (Fig 2), pollen presentation on each flower is strikingly different. Similarly, comparable nectar sugar masses per floral unit in *Echium vulgare* and *Cosmos bipinnatus* are presented in very different floral structures, and are more accessible to long and short-tongued bees, respectively. In addition to variation in sugar quantity, floral nectars also vary in concentration [48,49,100] and composition [101–103] in ways that influence their accessibility and attractiveness to different flower visitors (e.g. [104]).

These considerations show that for specific pollinators assessment of the resources provided by planted meadows will need to focus on rewards provided by specific plants, rather than meadow-wide estimates. The approach we demonstrate here is easily modified to incorporate such specificity by weighting values per floral unit (e.g. by percentage composition of pollen essential amino acids), or by selective incorporation of floral types visited by specific visitor taxa.

### Floral resources at the meadow level

Whilst the fine-scale results here necessarily reflect the particular species of the two seed mixes used the underlying seasonal dynamics are likely to be general. All meadow types and sites showed a seasonal rise and fall of resources, with those at perennial sites rising earlier in the season as would be anticipated from their life history (i.e. energy storage in perennating structures). In general we would expect considerable site-to-site variation even for a single mix in a single city (Figs 4, 6, 7 and 9; S4 and S5 Figs), resource dominance by a characteristic subset of species at any given point in the season (Figs 5 and 8) and substantial resource supplementation by weed species particularly at the start of the season (Figs 5 and 8).

All sown meadow treatments produced substantially higher mean nectar and pollen resources than amenity grassland controls. For the seed mixes that we tested, perennial meadows outperformed annual meadows for both nectar and pollen (Tables 1 and 2), though resources per unit area were broadly comparable. Annual A1 and A2 meadows produced broadly similar resource levels, but with higher between-replicate variation in both composition and resource level in the A2 treatment. We attribute this to stronger recruitment in A2 replicates of perennial weeds from the seed bank. This suggests that, as with planted field margins in farmland [105], intensive site preparation may be required to predictably yield target seed mix species, with potential for increased cost. We also note that highest resource levels for control sites were recorded when weed species had had most time to recover from the last mow (as for Morningside Park in Edinburgh, in June 2013). The distinct NMDS mean axis score of Reading (Fig 3C and 3D) is probably a signal of a local drought in 2013 that resulted in lower floral abundances.

A striking feature of the floral mixes used is that despite their species richness (14 and 23 species in the annual and perennial seed mixes respectively; S1 Table), meadow-scale resources at any point in the flowering season were dominated by a maximum of five annual species (Figs 5B and 5C, 8B and 8C) and four perennial species (Figs 5A and 8A), with some between-city variation in the species involved (S4A and S4B Table). Perennial nectar and pollen production were dominated by *Leucanthemum vulgare* and *Echium vulgare* earlier in the year, *Daucus carota* later in the year, and *Achillea millefolium* throughout. Annual meadow nectar production was spread over a wider set of species, while pollen production was dominated later in the year by *Papaver rhoeas*, *Eschscholzia californica* and *Centaurea cyanus*. Differences between treatments were most marked where a particular species became superabundant, as for *Daucus carota* in Edinburgh perennial meadows. Even though per-floret resources for this species are very low, it made a dominant contribution to peak perennial meadow nectar production in this city (Fig 5).

In contrast, several species in each seed mix made very low contributions to meadow level resources UK-wide, such as *Galium verum* and *Trifolium pratense* in the perennial mix or *Nigella damascena* in the annual mix. This raises the question of whether low-contributing species could be excluded from seed mixes to reduce costs [21]. However, as discussed above, diversity in floral resource quality and floral functional groups is positively correlated with pollinator health, diversity and abundance. The same patterns apply for seeded wildflower meadows [97–99]. To maintain pollinator populations, suitable floral resources must be available throughout the colony cycle of social species (such as honeybees and bumblebees). Rare flower types that contribute little to meadow-level resources but extend the diversity of floral rewards and structures and so attract specific visitor taxa, or which provide resources at key points in the season, could significantly enhance the biodiversity value of flower assemblages [21,106]. In addition to biodiversity benefits, rare flower types that have high aesthetic impact may increase public feelings of wellbeing and hence the amenity value of planted meadows [107–109]. Finally, variation in the contributions made by each plant species to flower counts for a given meadow treatment (reflected in the dispersal of points for each treatment in Fig 3) underlines the need for seed mixes to incorporate redundancy in the combinations of floral morphologies and resource types they offer. Such redundancy needs to incorporate sensitivity of component species to factors such as varying climatic conditions and soil conditions between years and/or planting sites.

The value of flower meadow resources for bee populations ultimately depends on the impact of their resources on pollinator population dynamics. It is possible to put the daily resources provided by planted meadows into context by using published estimates of the pollen required for individual bee development [22,110]. Based on a pollen density of 1.2 kg/dm^3^ [111,112] and a 300m^2^ treatment meadow, at their peak the A1 annual meadows produced 0.06–0.12g (0.05–0.1ml) pollen/m^2^/day, or 18–36g pollen/meadow/day (Fig 9). The Edinburgh perennial meadows produced 0.54g (0.45ml) pollen/m^2^/day, or 162g/meadow/day. Using the estimate that production of a single worker *Apis mellifera* requires *ca*. 150mg of pollen [113], these pollen masses equate to 120–240 worker bee equivalents/day for annual A1 meadows, and 1080/day for Edinburgh perennial meadows during peak production periods. While pollen production was much lower outside peak periods, over the full season several meadows offering such levels of resource would contribute significantly to the ca. 20kg of pollen/year required by a honeybee colony [114].

For solitary bees (calculated from pollen volumes in [22] and using the same pollen density estimate as above), pollen mass per brood cell scales positively with adult dry mass [22], ranging from 5.2mg for very small species such as *Hylaeus punctulatissimus* (Colletidae), to 80mg for the females of larger species such as *Colletes daviesanus*. Taken at face value, these figures suggest that available pollen in planted meadows could provide the resources required to produce many solitary bees. However, as discussed above the high flower specificity of many solitary species means that the value of planted meadows for them is likely to be more sensitive to meadow floral species composition, and is best estimated on a species-by-species basis.

### Timing of floral resource provision

A consistent pattern across cities was the earlier flowering of perennial meadows than annuals. However, neither of our seed mix treatments provided any floral resources in March, April or May. This matters because many flower visitors (including honeybees, queen bumblebees, and solitary bees such as some *Andrena*, *Anthophora* and *Osmia* species) are active early in the year in urban habitats (e.g. [18,115–117]). None of the 40 annual mix meadows produced resources early enough to meet the requirements of early season bumblebees such as *B*. *pratorum* and *B*. *hortorum*, whose colonies in the UK reach peak colony size in June [115], or of early summer species such as *B*. *lapidarius*, *B*. *lucorum* and *B*. *terrestris* during their first phase of colony growth.

In both annual and perennial treatments, up to 100% of nectar and pollen resources early in the year were provided by native perennial weeds, particularly *Taraxacum* agg., *Ranunculus repens* and *Trifolium repens*. While potentially problematic for green space managers from the point of view of public acceptance of planted meadows, and notwithstanding the pollen quality issues for some species discussed above, these weeds are favoured floral sources for bees and butterflies through spring and early summer in urban environments [51,117–120] and more widely (e.g. [121–124]). As managed, the mixes we used have very limited potential for provision of resources early in the year. While earlier sowing of an annual mix can result in earlier flowering when weather conditions allow earlier germination, this comes with higher risks to green space managers of meadow failure through rotting or predation of seeds or frost damage to young seedlings. Provision of floral resources for early-season pollinators requires targeted addition of early-flowering species to seed mixes. Possible perennial mix additions that are attractive to bees (assuming that appropriate soil and light conditions for their growth exist, and with pollen percentage protein by mass where available) include *Anemone nemorosa* [31], *Corydalis* spp. [32,125], *Pulmonaria officinalis* [126,127], *Alkanna tinctoria* and *Symphytum* spp. (17.5% protein; [54]) [125,126], *Lamium album* and *Lamium purpureum* [119] and *Erysimum* spp. [119]. Many temperate woody species flower early in the year [128]. Incorporating flowering native *Corylus avellana* (32% protein [53]), *Crataegus monogyna*, *Malus sylvestris* (25.1% [129]), *Arbutus unedo*, and *Prunus* spp. (43.6% protein [53]), and ornamentals such as *Cotoneaster* spp., *Mahonia* and *Berberis* spp., into green space management strategies could enhance both amenity value and early season resource availability to bees [31,119,120,122,130–134]. Other spring-flowering trees that are important sources of pollen for urban social and solitary bees include *Acer* spp. (40% protein; [53]), *Betula* spp. (29% protein; [53]), *Fraxinus excelsior* (33.3% protein; [53]), *Populus* spp. (40% protein; [53]), *Quercus* spp. (30–40% protein; [53]), and *Salix* spp. (37–47% protein; [53]) [117,122,130,131,135]. Integration of woody species into estimates of resource availability for pollinators requires quantification at a larger habitat or landscape scale. Finally, bees are also able to harvest pollen from grasses [136]. Though present at relatively low levels in our planted meadow seed mixes, grasses are an important component of urban plantings whose contribution to pollen provision for pollinators should be quantified.

### Predictive statistical models of floral resource provision

Our statistical models allow for prediction of pollen rewards per flower from floral morphology, nectar and pollen resources per unit areas from flower counts. The relationships provide a very good fit to observed data (Fig 10, S3 Fig), and in principle could be used to predict pollen resource values where only floral morphology of floral count data exists. This general approach can be taken a step further by modelling of flower abundance over time for a given meadow treatment or site. We can estimate the total production of flowers (or floral resources) for a given treatment or meadow on a given day, or over a given period from the sowing date by integration of a function fitted to floral resource availability over time. An example is shown in S6 Fig. Whilst we deliberately restricted this analysis to one meadow type in one city, the implication is that there may be a characteristic spectrum of floral abundance (and therefore of nectar and pollen resources) for a vegetation type. While necessarily approximate, generation of such relationships for the established vegetation classification units of a region (e.g. NVC in Britain) could provide the means to infer temporal patterns in floral resource availability at a landscape level. A similar approach has recently been applied at the landscape level by Baude et al [57].

Further work is required to determine variation in the strength and form of these relationships across floras in response, for example, to variation in taxonomic composition or pollen: ovule ratio [61]. The similarity of relationships between flower counts and resources for the taxonomically disparate annual and perennial seed mixes used here suggests that the relationship between these measures at the meadow level may be general. In estimating floral resources per flower, we would recommend focus of human resources on direct measurement of pollen volumes for the most abundant taxa in the vegetation, with statistical prediction for rarer taxa as an option that is likely to reduce the magnitude of errors associated with small sample sizes.

## Conclusions

We provide the first detailed analysis of pollen and nectar resources per species and per meadow in exemplar annual and perennial flower seed mixes. We show how resources are contributed by a subset of species in each mix that changes through time, and that each mix contains some species that contribute very little when quantified in this way. We show that native weeds contribute significantly to meadow resources, particularly earlier in the year.

Understanding the overall value of urban flower meadows requires quantifying the impact of seed mix and weed species on (i) the associated abundance and diversity of flower visitors, (ii) other components of biodiversity through provision of food, shelter or nest sites, such as non-pollinating insects and insectivorous or seed-eating birds, and (iii) the value of urban meadows to human populations. In addition to floral resources, flower-visiting insects also require nest sites (bees), food plants (butterflies) or other larval resources (aphidophagous hoverflies), and enrichment of urban biodiversity requires consideration of each of these requirements.

For green space managers, key considerations will include public attitudes towards change in land use, cost of establishment, and maintenance of the meadows. The latter will be dependent upon the type of mix used and aim of the planting, careful matching of seed mixes to local site conditions, and management requirements (frequency of weeding, litter removal). Seed provenance (whether native *or* non-native) can be an important consideration. We found some non-native species in annual seed mix used to provide substantial resources, and there is evidence that, in an urban context, non-native species can provide useful resources to pollinators [137].

Burgeoning interest in urban meadows and seed mix production provide an excellent scientific opportunity to monitor both drivers and outcomes of this form of biodiversity intervention. Though flower surveys are labour-intensive, they can provide a good statistical basis for floral resource estimation. Statistical models could be developed for any location where there is an interest in diagnosing the performance of a seed mix or stand of vegetation, and may make it possible to track changes in resource provision over time using published quantitative floral surveys, such as the UK terrestrial NVC and aquatic WFD surveys or [62] urban botanical surveys.

## Supporting Information

S1 Fig Effects of sampling effort, in terms of numbers of 1m^2^ quadrats sampled, on estimates of the mean and variance of meadow nectar sugar mass per day. Coloured plots show nine runs of sampling data from 40 randomised quadrats, sampled without replacement. (A) Inch Park A2 annual meadow, Edinburgh, on 29/08/2012 (n = 99 quadrats sampled, overall mean of 4610.5 mg/day). (B) Cairntows A2 annual meadow, Edinburgh on 02/08/2012. (n = 77 quadrats sampled, overall mean 5050.5 mg/day).
(PDF)

S2 Fig Effects of sampling effort, in terms of numbers of 1m^2^ quadrats sampled, on estimates of the mean and variance of meadow pollen volume per day. Coloured plots show nine runs of sampling data from 40 randomised quadrats, sampled without replacement. (A) Inch Park A2 annual meadow, Edinburgh, on 29/08/2012 (n = 99 quadrats sampled, overall mean 45.25 ml/day). (B) Cairntows A2 annual meadow, Edinburgh on 02/08/2012. (n = 77 quadrats sampled, overall mean 47 ml/day).
(PDF)

S3 Fig The relationship between laboratory measures of pollen volume /floral unit and model-fitted values. The table below shows the coefficients for the best-fit model, in which the missing anther size category (large) corresponds to the intercept value. The plot shows the relationship between lab measured and modelled values for the test data (red) and the validation data (blue). The fitted line shows x = y, to which both datasets have a very good fit.
(PDF)

S4 Fig Seasonal patterns in nectar sugar mass/m^2^/day for each site in (a) Bristol, (b) Leeds, and (c) Reading. Point values shown are means, with 95% confidence intervals shaded. Low values for Victoria Park (perennial, Bristol) Tames Promenade (A1, Reading) and Portman Road west (A1 Reading) were associated with management problems, and these sites were excluded from statistical analyses.
(PDF)

S5 Fig Seasonal patterns in pollen volume per m^2^/day for each site in (a) Bristol, (b) Leeds, and (c) Reading. Point values shown are means, with 95% confidence intervals shaded. Low values for Victoria Park (perennial, Bristol) Tames Promenade (A1, Reading) and Portman Road west (A1 Reading) were associated with management problems, and these sites were excluded from statistical analyses.
(PDF)

S6 Fig Statistical modelling of the relationship between time (number of days after planting) and mean floral unit density per quadrat for Edinburgh A1 meadows. (PDF)

S7 Fig Comparison of species nectar sugar mass values per floral unit for different populations of the same species, generated using the same assay protocols by Edinburgh and by Baude et al. (2016) [57]. (a) Plot showing Edinburgh (x axis) and Baude et al (2016) (y axis) values for per-species nectar sugar mass, relative to a line with equation x = y. Error bars are ± 95% confidence intervals. (b) Bar plot in which the two values for individual species are more easily visible. All shared species for which directly comparable values are available (all non-Asteraceae) are included.
(PDF)

S1 File Meadow seeding and maintenance protocols. (DOCX)

S2 File Nectar quantification methods. (DOCX)

S3 File Pollen and floral unit longevity quantification methods. (DOCX)

S1 Table Floral resources by species. Common names, resource levels/day, and floral longevities for the species in the annual and perennial seed mixes used in the study, and associated weeds. Total pollen volumes per flower (rather than volumes/day) and their errors are given in S3 Table. Species for which we did not estimate resources (given prioritisation of common species for resource estimation) are marked 'na'. Sample sizes (n) for estimation of each resource type and species are given.
(XLS)

S2 Table Sample site location information. Study site information for each of the four cities (Bristol, Edinburgh, Leeds and Reading). Site location information includes the location name (most often a park or school), street name location, and UK vice county (VC) name and number. The column ‘Block’ refers to the location of each site within 5 geographically grouped sampling blocks in each city.
(DOCX)

S3 Table Data used to model the relationship between floral morphology (anther size class, mean stamen number per floret or flower) and measured mean pollen volume per flower or floret. Data are provided on standard errors of mean measured values, and model fitted values (for model, see S3 Fig). We provide two measures of the mismatch between measured and model values—the absolute difference in values, and these differences as a percentage change in the measured value.
(XLS)

S4 Table Univariate analysis outputs of mvabund, showing species contributing most significantly to variation in meadow composition (incorporating both presence absence and relative abundance of floral units across surveys). The species listed in each table comprise those with an individual p ≤ 0.05 or the set of species contributing cumulatively to 50% of the explained variance, whichever was greater. The status of each species is shown as sown (i.e. in a seed mix) or weed. The right hand column shows the direction of trends in abundance of each species across cities.
(DOCX)

S5 Table Widely available pollinator seed mixes and their constituent plant species. A cross in the row for a plant species indicates presence in a given seed mix. The Count column at right shows the total number of mixes in which a given species is represented. The most commonly shared species include *Centaurea cyanus*, *Leucanthemum vulgare* (5 mixes), and *Centaurea nigra*, *Daucus carota*, *Lotus corniculatus*, *Silene dioica*, and *Trifolium pratense* (4 mixes).
(XLSX)
