# Supplementary material for: Food for Pollinators: Quantifying the Nectar and Pollen Resources of Urban Flower Meadows
Source: PLoS One. 2016 Jun 24;11(6):e0158117. doi: 10.1371/journal.pone.0158117 (PMC4920406; doi:10.1371/journal.pone.0158117)

Figure S1. Effects of sampling effort, in terms of numbers of 1m^2 quadrats sampled, on estimates of the mean and variance of meadow nectar sugar mass per day. Coloured plots show nine runs of sampling data from 40 randomised quadrats, sampled without replacement. (A) Inch Park A2 annual meadow, Edinburgh, on 29/08/2012 (n=99 quadrats sampled, overall mean of 4610.5 mg/day). (B) Cairntows A2 annual meadow, Edinburgh on 02/08/2012. (n= 77 quadrats sampled, overall mean 5050.5 mg/day).

A. Inch Park, Edinburgh.

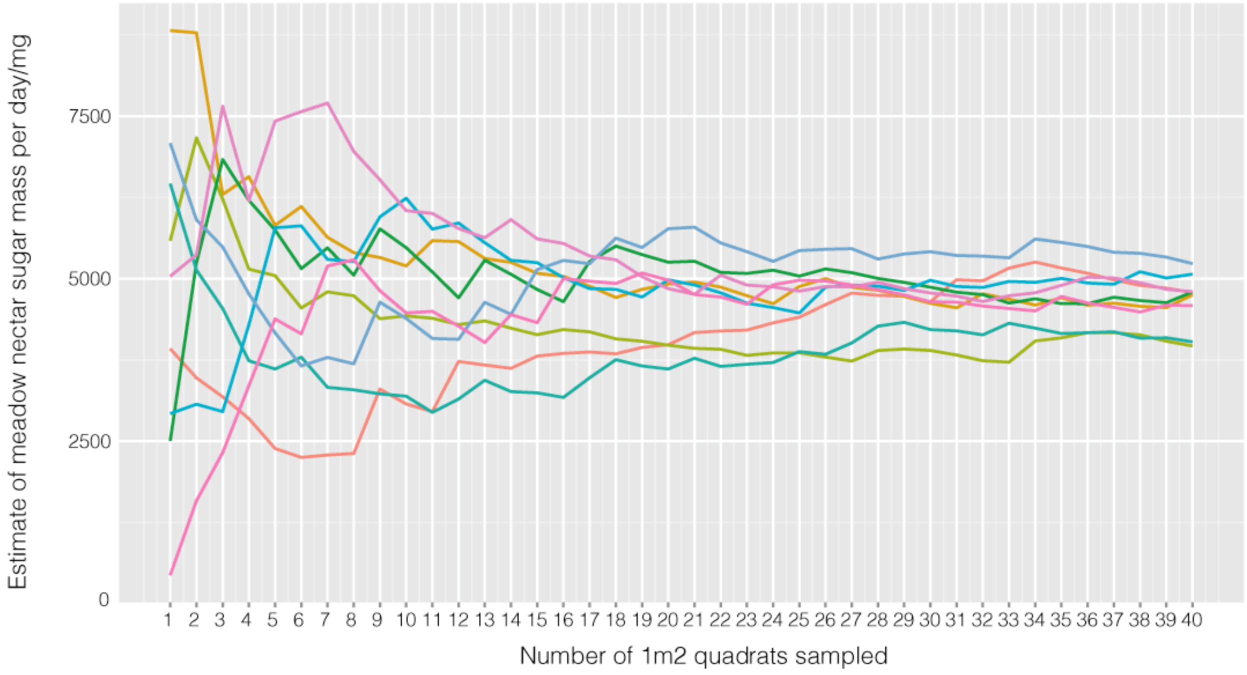

B. Cairntows Park, Edinburgh

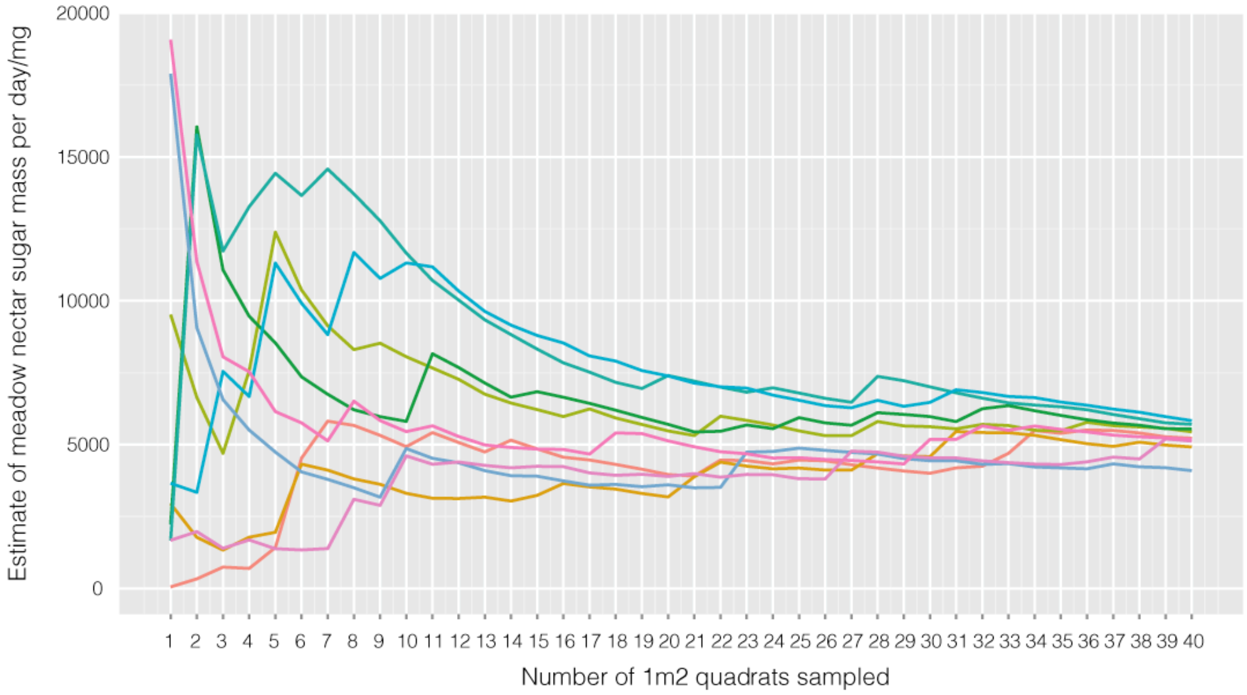

Supplement: S1 Fig — Coloured plots show nine runs of sampling data from 40 randomised quadrats, sampled without replacement. (A) Inch Park A2 annual meadow, Edinburgh, on 29/08/2012 (n = 99 quadrats sampled, overall mean of 4610.5 mg/day). (B) Cairntows A2 annual meadow, Edinburgh on 02/08/2012. (n = 77 quadrats sampled, overall mean 5050.5 mg/day). (PDF) [file pone.0158117.s001.pdf]
