# Supplementary material for: Food for Pollinators: Quantifying the Nectar and Pollen Resources of Urban Flower Meadows
Source: PLoS One. 2016 Jun 24;11(6):e0158117. doi: 10.1371/journal.pone.0158117 (PMC4920406; doi:10.1371/journal.pone.0158117)

Figure S2. Effects of sampling effort, in terms of numbers of $1m^2$ quadrats sampled, on estimates of the mean and variance of meadow pollen volume per day. Coloured plots show nine runs of of sampling data from 40 randomised quadrats, sampled without replacement. (A) Inch Park A2 annual meadow, Edinburgh, on 29/08/2012 (n= 99 quadrats sampled, overall mean 45.25 ml/day). (B) Cairntows A2 annual meadow, Edinburgh on 02/08/2012. (n= 77 quadrats sampled, overall mean 47 ml/day).

A. Inch Park, Edinburgh.

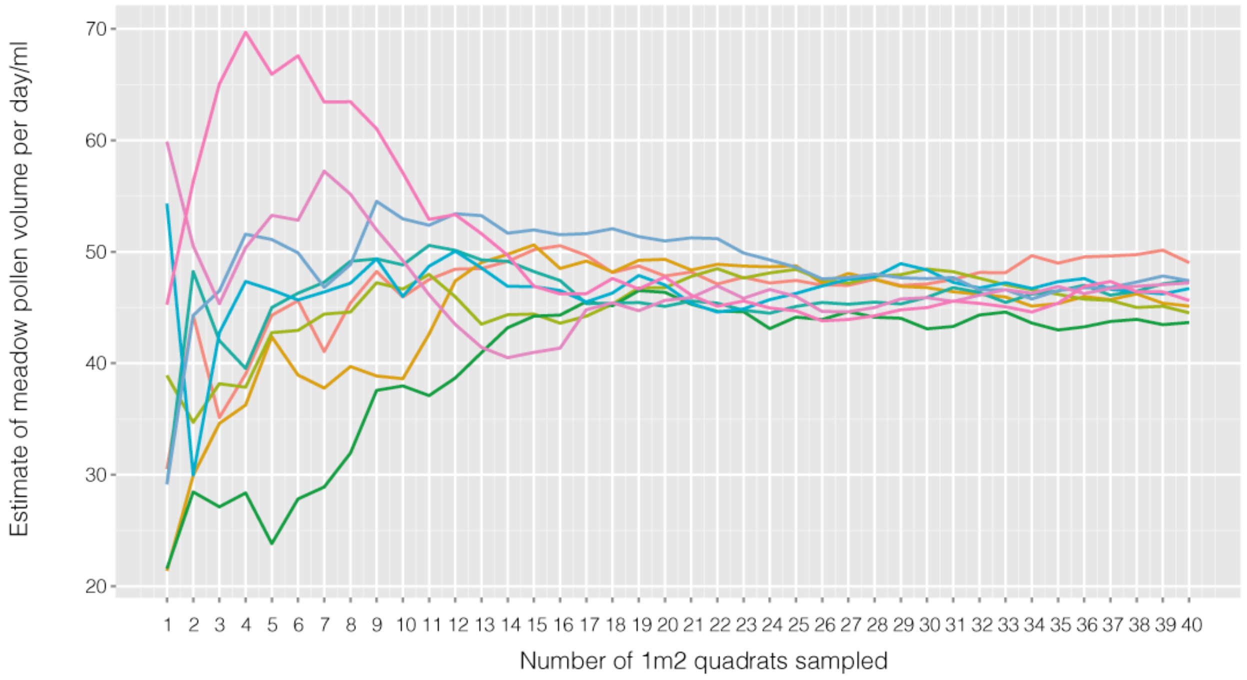

B. Cairntows Park, Edinburgh

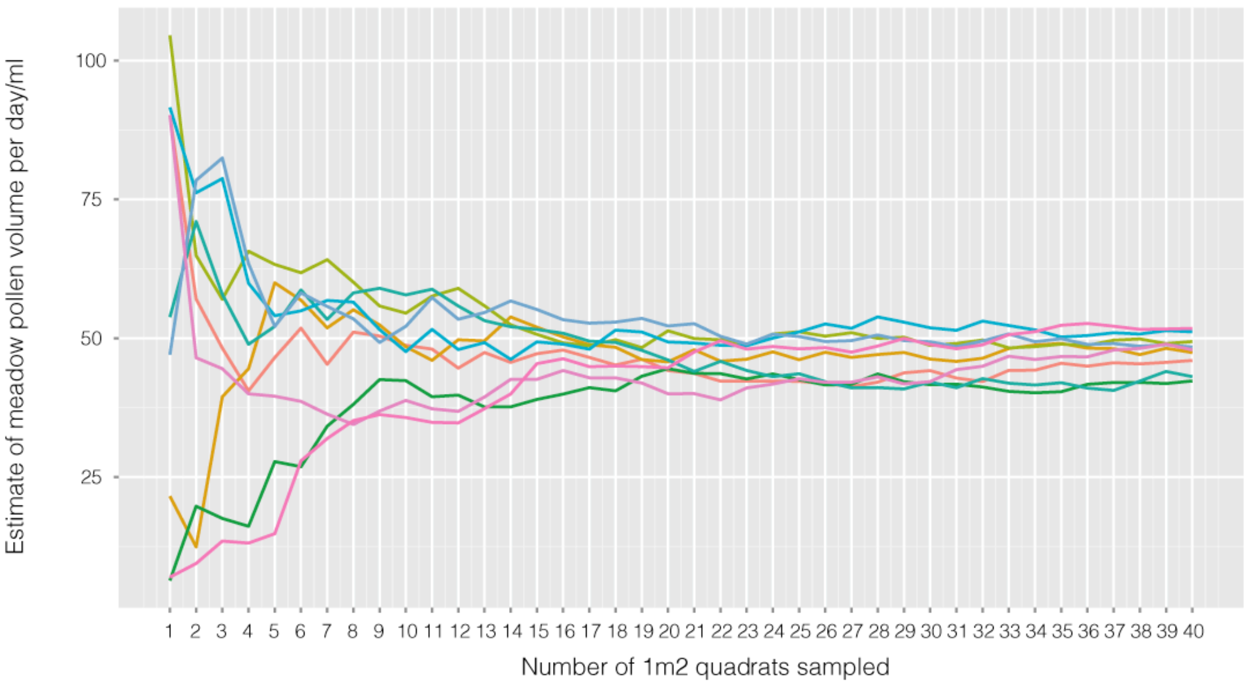

Supplement: S2 Fig — Coloured plots show nine runs of sampling data from 40 randomised quadrats, sampled without replacement. (A) Inch Park A2 annual meadow, Edinburgh, on 29/08/2012 (n = 99 quadrats sampled, overall mean 45.25 ml/day). (B) Cairntows A2 annual meadow, Edinburgh on 02/08/2012. (n = 77 quadrats sampled, overall mean 47 ml/day). (PDF) [file pone.0158117.s002.pdf]
