# Supplementary material for: Food for Pollinators: Quantifying the Nectar and Pollen Resources of Urban Flower Meadows
Source: PLoS One. 2016 Jun 24;11(6):e0158117. doi: 10.1371/journal.pone.0158117 (PMC4920406; doi:10.1371/journal.pone.0158117)

Figure S3. The relationship between laboratory assays and model-fitted values of pollen volume /floral unit. The table below shows the coefficients for the best-fit model, in which the missing anther size category (large) is the dummy variable. The plot shows the relationship between lab-measured and modelled values for the test data (red, n = 59) and the validation data (blue, n = 43). The fitted line shows x=y, to which both datasets show a good fit.

lm model specification for the test data:
$\log_e$ pollen volume per flower ~ 1/number of stamens/flower + anther.size

Coefficients:

	Estimate	Std. Error	t value	p value
Intercept	2.3350	0.6029	3.873	0.0003
1/stamens per flower	-8.9835	1.9868	-4.521	<<0.0001
anther.size medium	-3.2777	0.6475	-5.062	<<0.0001
anther.size small	-5.5904	0.6617	-8.448	<<0.0001
anther.size tiny	-4.4862	0.6653	-6.743	<<0.0001

Residual standard error: 1.149 on 54 degrees of freedom
Multiple R-squared: 0.723, Adjusted R-squared: 0.7025
F-statistic: 35.23 on 4 and 54 DF, p-value <<0.0001

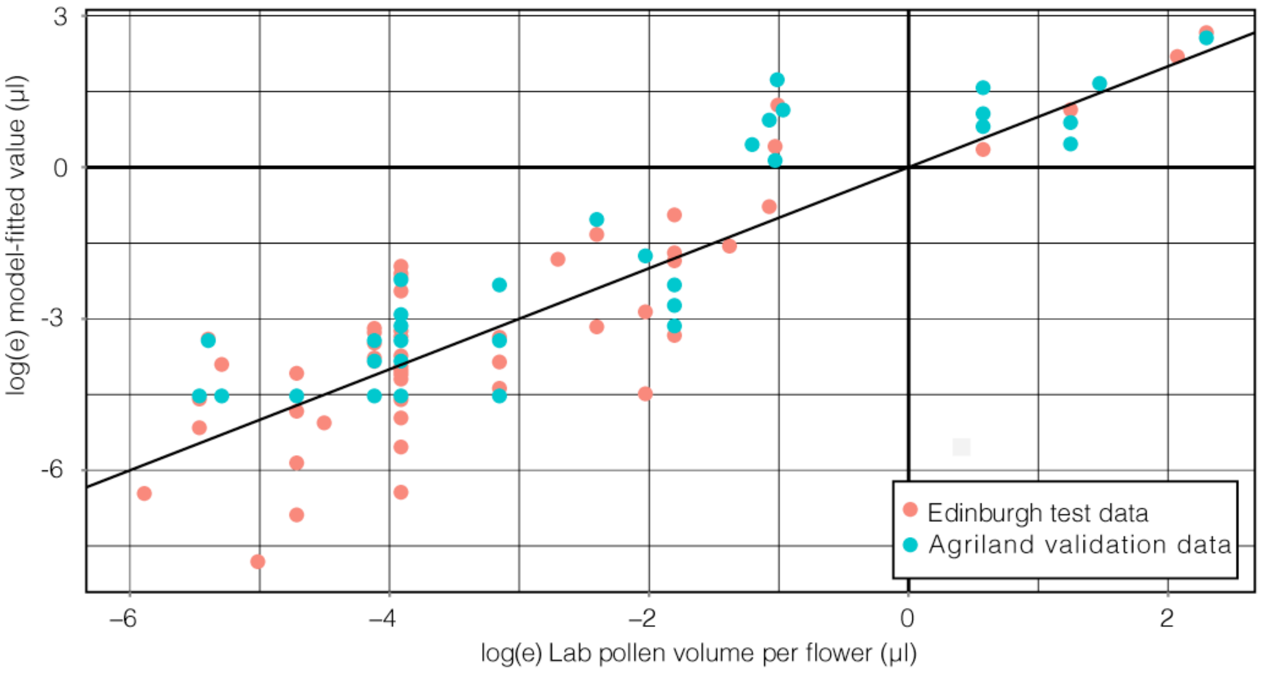

Supplement: S3 Fig — The table below shows the coefficients for the best-fit model, in which the missing anther size category (large) corresponds to the intercept value. The plot shows the relationship between lab measured and modelled values for the test data (red) and the validation data (blue). The fitted line shows x = y, to which both datasets have a very good fit. (PDF) [file pone.0158117.s003.pdf]
