# Supplementary material for: Food for Pollinators: Quantifying the Nectar and Pollen Resources of Urban Flower Meadows
Source: PLoS One. 2016 Jun 24;11(6):e0158117. doi: 10.1371/journal.pone.0158117 (PMC4920406; doi:10.1371/journal.pone.0158117)

Figure S5. Seasonal patterns in pollen volume per m^2/day for each site in (a) Bristol, (b) Leeds, and (c) Reading. Point values shown are means over 7x 1m^2 quadrats, with 95% confidence intervals shaded.

(a) Bristol: Perennial

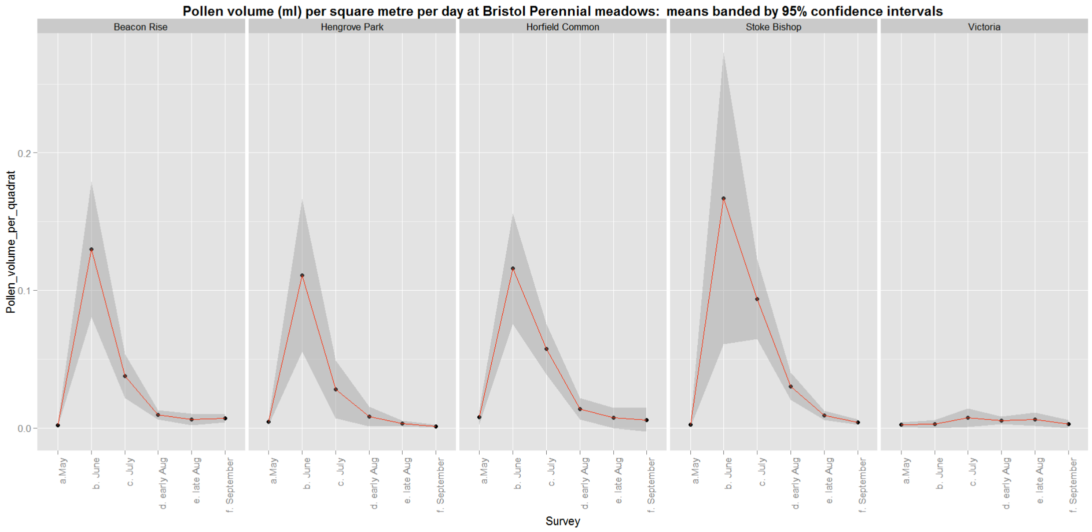

Annual A1

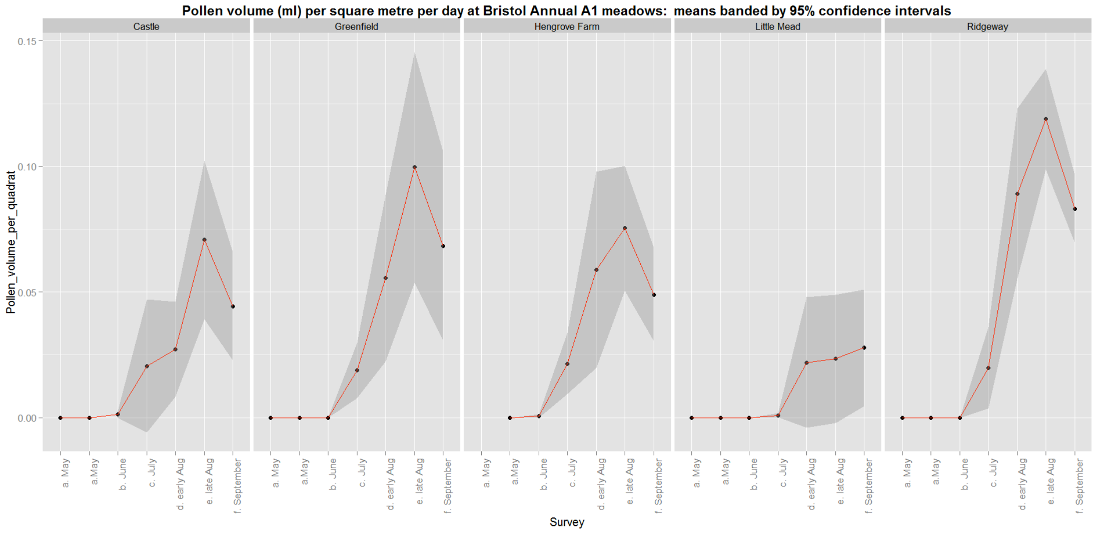

Annual A2

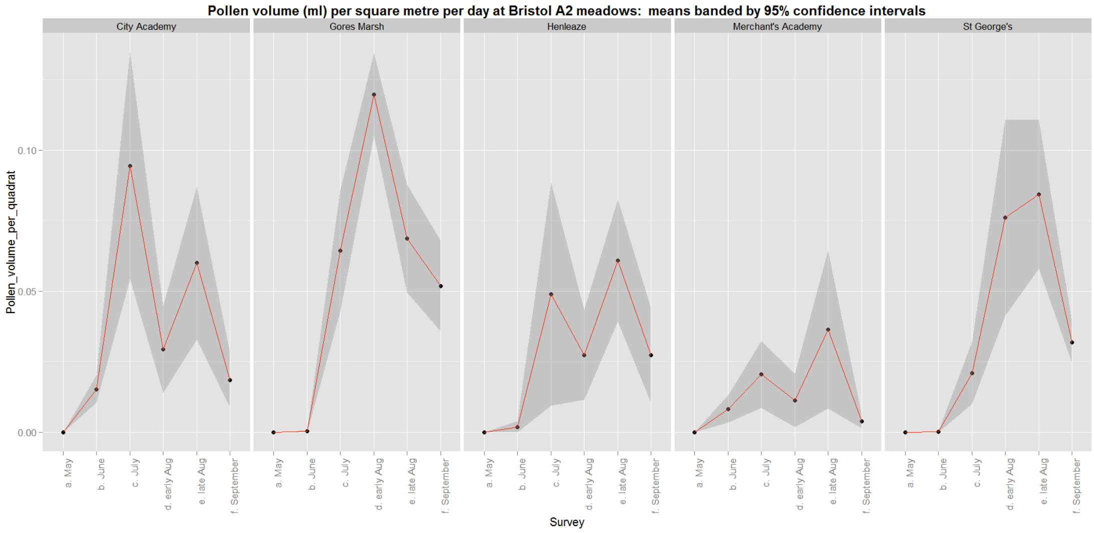

Figure S5 (continued). Seasonal patterns in pollen volume per m^2/day for each site.
(b) Leeds
Perennial

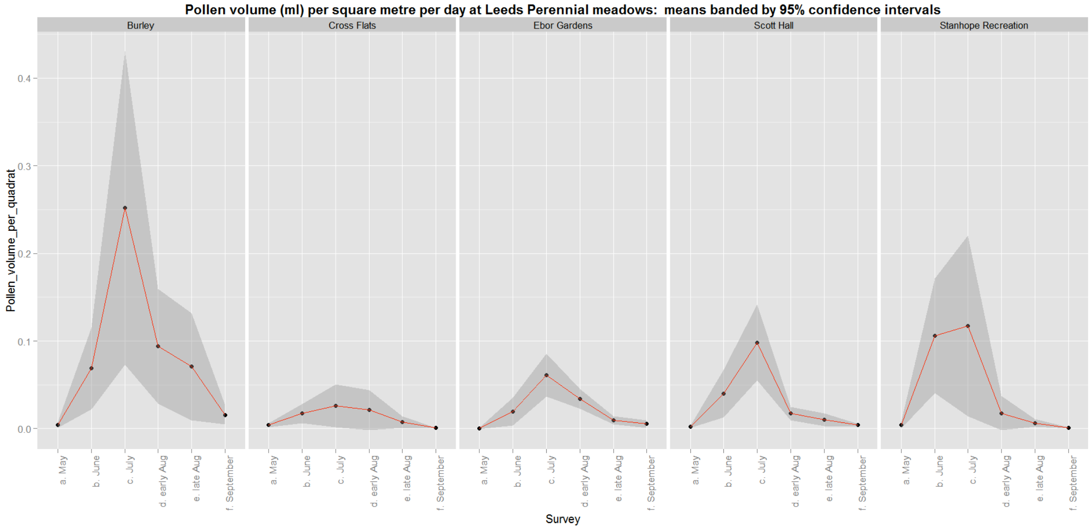

Annual A1

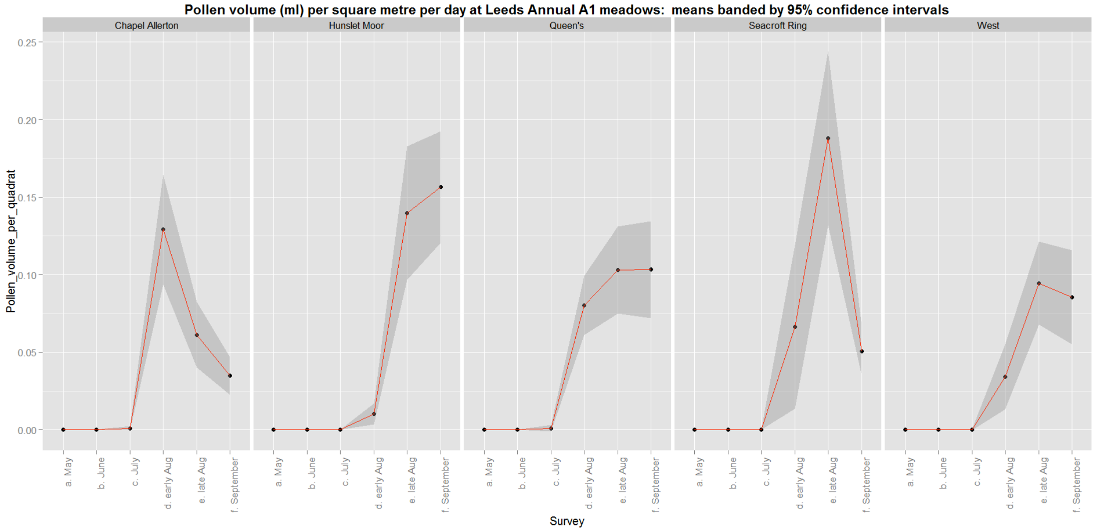

Annual A2

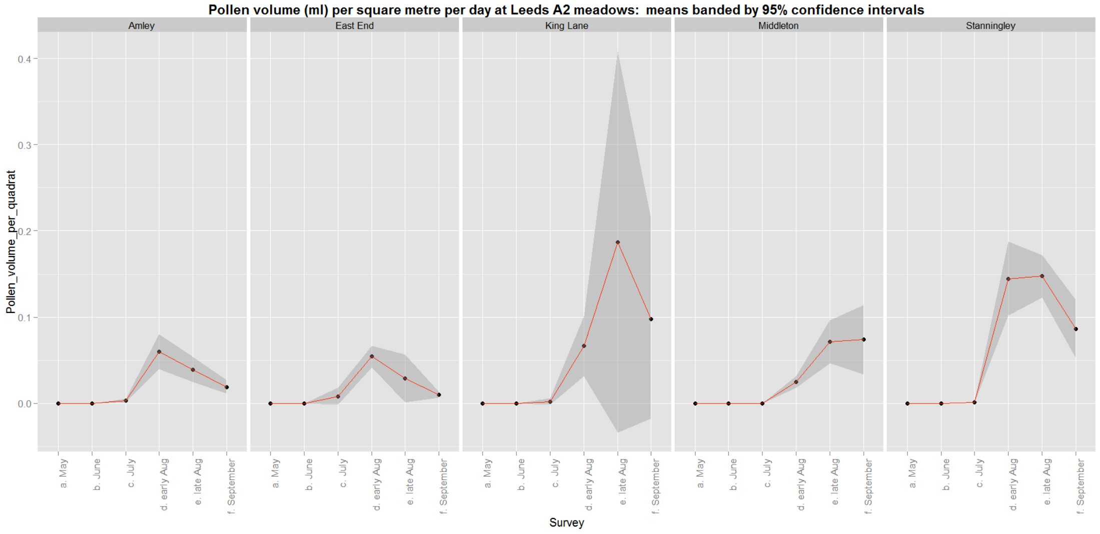

Figure S5 (continued). Seasonal patterns in pollen volume per m^2/day for each site.
(c) Reading
Perennial

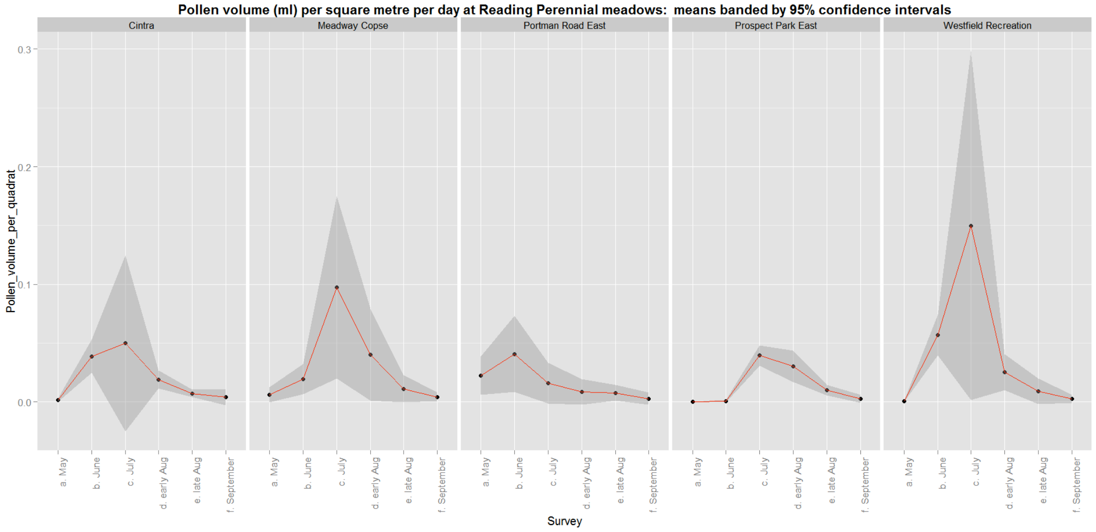

Annual A1

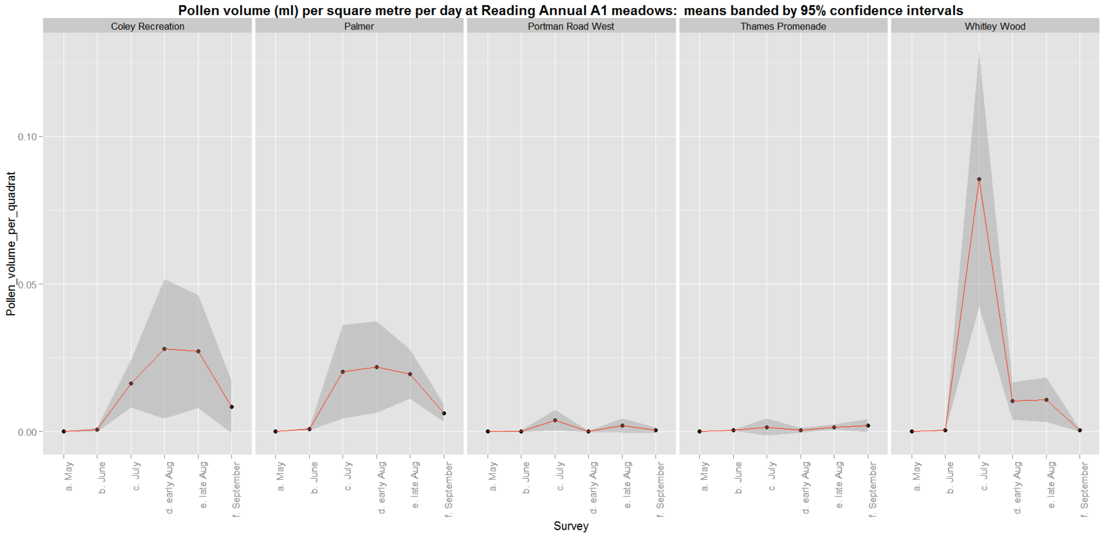

Annual A2

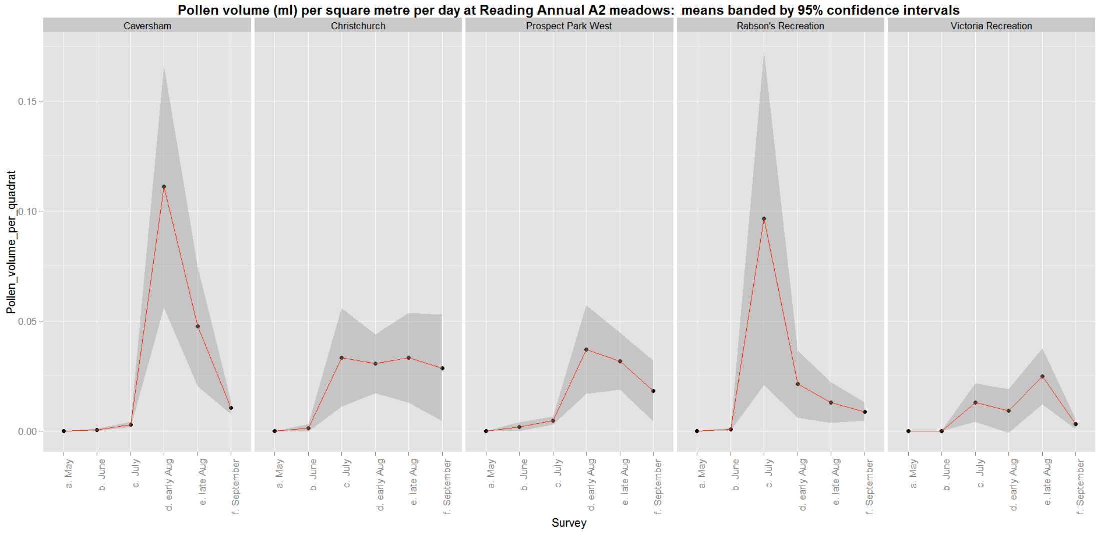

Supplement: S5 Fig — Point values shown are means, with 95% confidence intervals shaded. Low values for Victoria Park (perennial, Bristol) Tames Promenade (A1, Reading) and Portman Road west (A1 Reading) were associated with management problems, and these sites were excluded from statistical analyses. (PDF) [file pone.0158117.s005.pdf]
