# Supplementary material for: Food for Pollinators: Quantifying the Nectar and Pollen Resources of Urban Flower Meadows
Source: PLoS One. 2016 Jun 24;11(6):e0158117. doi: 10.1371/journal.pone.0158117 (PMC4920406; doi:10.1371/journal.pone.0158117)

Figure S6: Statistical modelling of the relationship between time (number of days after planting) and mean floral unit density per quadrat of Edinburgh A1 meadows. Values for the two pilot studies of 2012 are superimposed (in red) to assess replicability of patterns between years. Any zero scores, and values for meadows < 70 days old, were excluded.

A polynomial curve (shown below) was fitted to the data using a simple rule of improving fit while minimising negative values. For these data this approach supported a fourth order polynomial equation ($-109057 + 4956.938x - 83.82101x^2 + 0.6232503x^3 - 0.00170689x^4$ $p = 0.009$; Adj $R^2 = 0.56$), shown fitted to the total dataset. The integral of the fitted function allows us to infer floral abundance for a given day or period of days in the season.

As an example up to the first week of August for an A1 meadow sown on 1st May, the integral :

$$\int_{92}^{99} -109057 + 4956.938x + -83.82101x^2 + 0.6232503x^3 + -0.00170689x^4 \, dx$$

$$= 5117.318$$

$$= 5100 \text{ floral units per m}^2$$

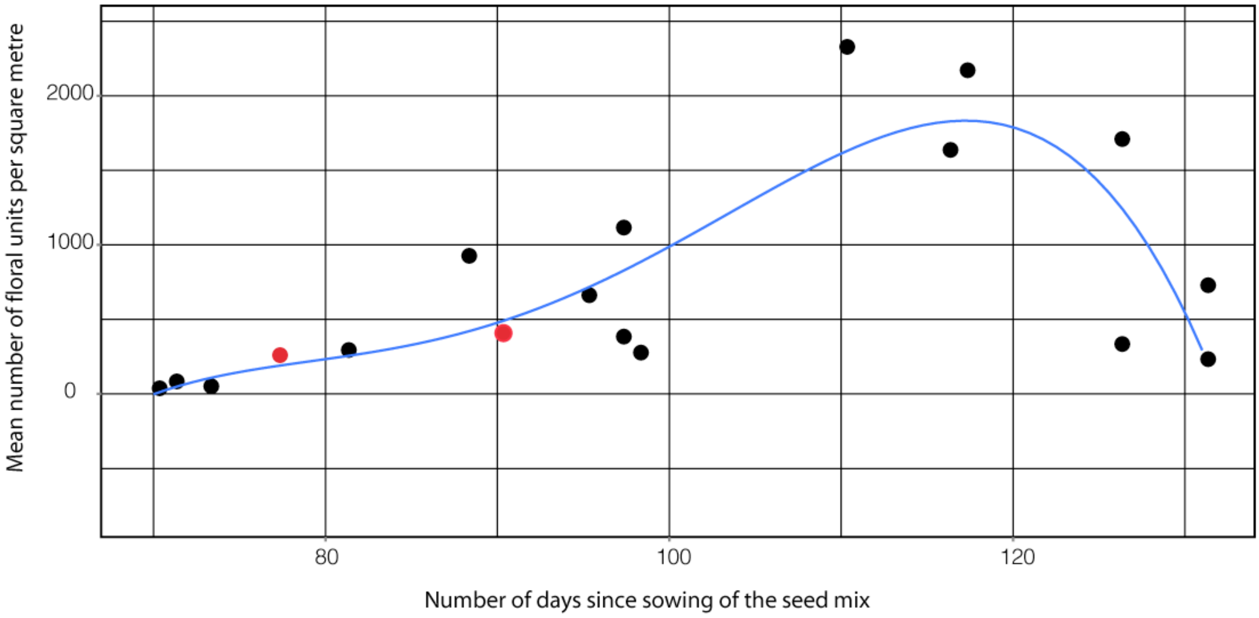

Supplement: S6 Fig — (PDF) [file pone.0158117.s006.pdf]
