# Supplementary material for: Food for Pollinators: Quantifying the Nectar and Pollen Resources of Urban Flower Meadows
Source: PLoS One. 2016 Jun 24;11(6):e0158117. doi: 10.1371/journal.pone.0158117 (PMC4920406; doi:10.1371/journal.pone.0158117)

Figure S7: Comparison of species nectar sugar mass values per floral unit for different populations of the same species, generated using the same assay protocols by Edinburgh and by Baude et al. (2016) (57). (a) Plot showing Edinburgh (x axis) and Baude et al. (y axis) values for per-species nectar sugar mass, relative to a line with equation x=y. Error bars are ± 95% confidence intervals. (b) Bar plot in which the two values for individual species are more easily visible. All shared species for which directly comparable values are available (all non-Asteraceae) are included.

(a)

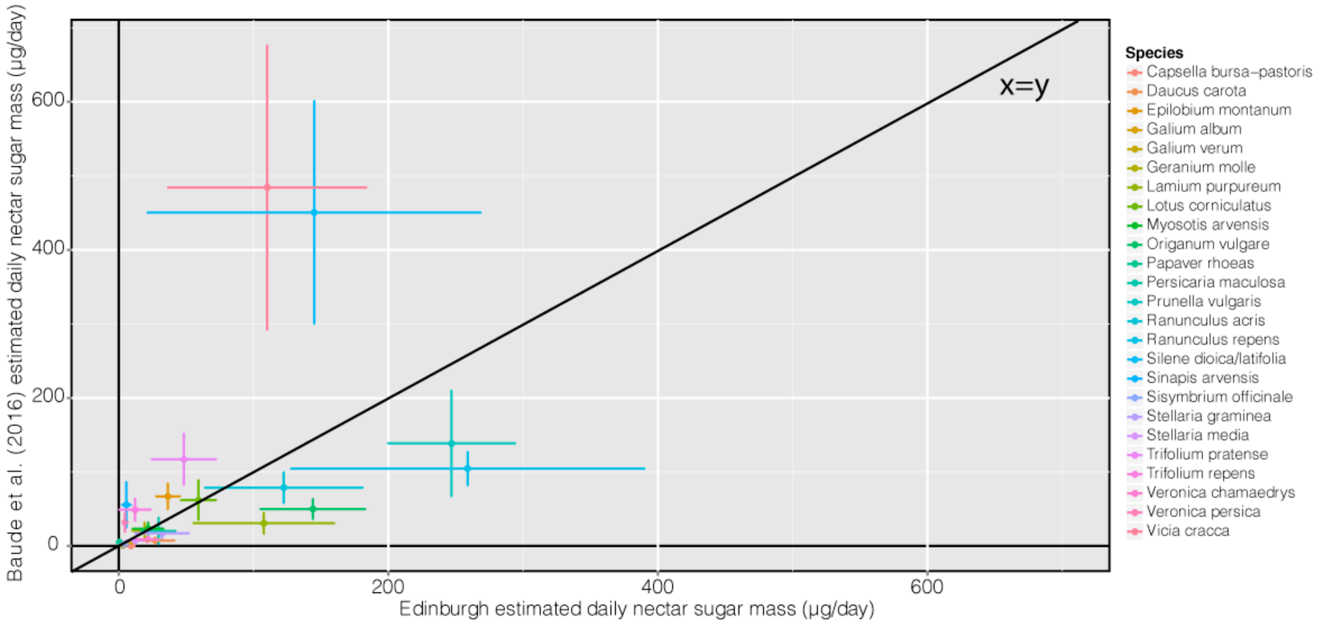

(b)

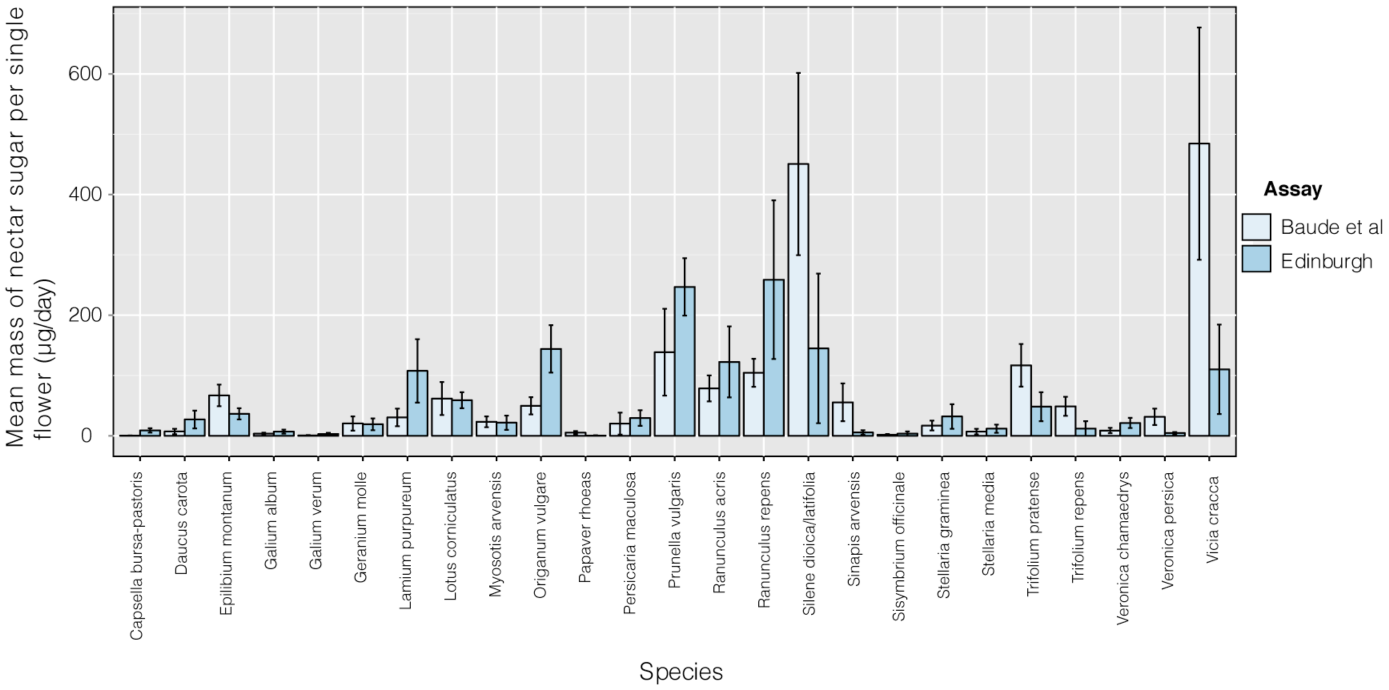

Supplement: S7 Fig — (a) Plot showing Edinburgh (x axis) and Baude et al (2016) (y axis) values for per-species nectar sugar mass, relative to a line with equation x = y. Error bars are ± 95% confidence intervals. (b) Bar plot in which the two values for individual species are more easily visible. All shared species for which directly comparable values are available (all non-Asteraceae) are included. (PDF) [file pone.0158117.s007.pdf]
