# Supplementary material for: Food for Pollinators: Quantifying the Nectar and Pollen Resources of Urban Flower Meadows
Source: PLoS One. 2016 Jun 24;11(6):e0158117. doi: 10.1371/journal.pone.0158117 (PMC4920406; doi:10.1371/journal.pone.0158117)
Supplement: S1 File — (DOCX) [file pone.0158117.s008.docx]

**File S1** Meadow establishment and maintenance protocol

**Annual meadows:**

The A2 annual meadows were sown at the end of May 2012 and re-sown at the beginning of May 2013, at the same time as the A1 annual meadows. Ground preparation involved spraying off the existing grass with Glyphosate and rotovating the soil to produce a seedbed for sowing. Seeds were sowed at a density of 3 g/m^2^, and were mixed with fine sand before sowing to facilitate an even spread. Weeds taller than the surrounding meadow were removed either by cutting to ground level or carefully pulling the whole plant out, including the roots, to prevent regrowth. Weeds that could not be reached from the meadow margin were not removed, to avoid trampling the seed mix plants. The most troublesome weeds were Sow-thistles (*Sonchus* spp.), Thistles (*Cirsium* spp.), Docks (*Rumex crispus* and *R. obtusifolius*) and Goosefoots (*Chenopodium* spp.). At the end of 2012 the meadows were mowed and the cuttings removed to keep the meadow sites looking neat. Site preparation, sowing and maintenance of A1 and A2 annual meadow sites followed this same procedure in the spring of 2013.

**Perennial meadows:**

Perennial meadow sites were prepared for sowing in the same way as annual meadows. The perennial mix used could be sown in either autumn or spring. In 2012 the ground was too dry in March for preparation and wet weather in April further slowed ground preparation. Meadows were sown at the end of May, and mowed with removal of cuttings in September 2012. Seeds were sown at a density of 1.5 g/m^2^, and were mixed with fine sand before sowing to facilitate an even spread. Weeds taller than the surrounding meadow were removed either by cutting to ground level or carefully pulling the whole plant out, including the roots, to prevent regrowth. The most troublesome weeds were Sow-thistles (*Sonchus* spp.), Thistles (*Cirsium* spp.) and Dandelion (*Taraxacum* agg.).

All meadows were inspected in April 2013. Any sites with vegetation growth above 10 cm in height were mown back to 5 cm. Two sites in Edinburgh (Saughton and St Katharine’s parks) that were badly affected by grass regrowth were treated with the gramicide ‘Fusilade’ in late May 2013.
