# Supplementary material for: Food for Pollinators: Quantifying the Nectar and Pollen Resources of Urban Flower Meadows
Source: PLoS One. 2016 Jun 24;11(6):e0158117. doi: 10.1371/journal.pone.0158117 (PMC4920406; doi:10.1371/journal.pone.0158117)
Supplement: S2 File — (DOCX) [file pone.0158117.s009.docx]

Supplemental File S2 **Quantification of floral nectar sugar per flower per day**

Because nectar secretion rate varies through the day (1), nectar sugar rewards per flower were estimated from that which was allowed to accumulate in sampled flowers over 24 hours. This sugar measure represents the gross secretion rate (i.e. the balance between sugar secretion and any resorption) and may underestimate actual sugar secretion (1). Because the nectar resource can vary among flowers on a plant, and between plants within species, wherever possible we sampled up to two flowers per plant from 11 plants for species sampled as individual flowers, and two florets from each of five capitula (each sampled from a different plant) for Asteraceae (Table S1).

Some flowers bagged in preparation for sampling (see below) were vandalised, reducing sample sizes below desired targets. Each flower was sampled once only. As nectar volumes are often very small, and as cutting the flower can modify the nectar quantity, concentration and/or composition, we sampled flowers individually in the field.

Field sampling required two days per sample, and was restricted to periods of dry weather to avoid nectar dilution or rinsing by rain. Sampling was at the level of individual flowers for all species other than Asteraceae, for which capitula were sampled (we distinguish between these as required below). These procedures are available from the authors on request in the form of a detailed protocol.

**Flower bagging (day 1)**

Flowers to be sampled were marked by slipping short cut sections of plastic drinking straw around each stem. The ringed flowers were then covered with a bag made of nylon 1x1 mm mesh (following 1) to exclude insect visitors. Each bag was closed around the plant with string.

**Nectar sampling (day 2)**

Floral nectar was collected by one of two protocols. For those species in which visible nectar could be obtained, nectar was sampled directly using microcapillary tubes (VWR International), starting with a 1 µl microcapillary and using larger volumes where available nectar allowed.

For each flower, we used as many capillaries as were necessary to empty the flower. The microcapillaries from a single flower were placed in the same labelled tube, which was immediately placed on ice to reduce any nectar evaporation and/or sugar alteration.

If the sampled flower or floret yielded no visible column of nectar in the smallest volume microcapillary tube, we rinsed the flower with 1µl of distilled water injected with a 2 µl micropipette, as close as possible to the location of the nectaries. After one minute, the injected 1 µl was collected from the flower with microcapillary tubes. The microcapillaries from each flower were placed in a tube, recorded as the first rinsing (A), and immediately placed on ice. Because it can be difficult to extract all sugar in a single rinse, we carried out a second rinse of each sampled flower/floret, as above, placing the resulting filled microcapillaries in a second tube, recorded as the second rinsing (B) and immediately put on ice. We recognise that this procedure is a necessary compromise between the need to sample low volumes of crystalline or very viscous nectar, and the risk that addition of water could result in release of sugars not normally accessible to flower visitors (1).

**Nectar measurement (volume and sugar content) in the lab**

Nectar sugar per flower was calculated as the product of nectar volume x sugar mass/unit nectar volume, and was calculated in a similar way whether using directly sampled nectar or floral rinses. The procedures below were carried out separately for rinses A and B where this procedure was used, and the sugar mass estimates combined to give a value per flower.

(i) Nectar volume

The total volume of nectar or rinse from each flower was calculated by using calipers to measure the length of the nectar columns in the microcapillary tubes. Total volume/flower was obtained using (total measured nectar column length/length of a single microcapillary) x microcapillary unit volume.

(ii) Nectar concentration

Nectar concentration was measured using a sucrose refractometer (Bellingham and Stanley, UK; 0-50° and 45-80°Brix) modified by the makers to accommodate low sample volumes. 1°B corresponds to 1 g of sucrose per 100g of solution. Because concentration measurement is temperature-dependent, we calibrated the refractometers for use at a lab temperature of 20°C using distilled water and sucrose concentrations of known sucrose mass/100g solution. Samples were removed from ice and allowed to warm to lab temperature before measurement. After each measurement, we cleaned the refractometer prism with distilled water and optical paper.

Nectar sugar concentrations were then transformed from °Brix to mass/unit volume of solution, following Prys-Jones & Corbet (2).

1. Corbet, S.A. (2003). Nectar sugar content: estimating standing crop and secretion rate in the field. Apidologie 34 (2003) 1–10 DOI: 10.1051/apido:2002049
2. Prys-Jones, O. E., & Corbet, S. A. (1991). *Naturalists’ Handbooks 6: Bumblebees*. Slough: Richmond Publishung CO. Ltd.
