# Supplementary material for: Food for Pollinators: Quantifying the Nectar and Pollen Resources of Urban Flower Meadows
Source: PLoS One. 2016 Jun 24;11(6):e0158117. doi: 10.1371/journal.pone.0158117 (PMC4920406; doi:10.1371/journal.pone.0158117)
Supplement: S3 File — (DOCX) [file pone.0158117.s010.docx]

Supplemental File S3 **Quantification of floral pollen volume per flower**

Pollen volume per flower for each species was estimated as the product of (number of anthers per flower) x (number of pollen grains per anther) x (the volume of a pollen grain). For each plant species sampled, we aimed to estimate pollen rewards per flower from at least 5 individual plants (see Table S1). The number of flowers sampled per replicate plant varied, depending on the number of flowers required to produce a measurable pollen volume (see below). Across all 64 species sampled, we sampled pollen from a total of 15,925 individual flowers.

Flowers for pollen sampling were collected as buds that were about to open. Plant stems were cut close to the ground and placed in water for return to the lab. All flowers that were open at the time of sampling were removed from collected plant material. Cut stems were maintained at lab temperature under natural light, in areas without drafts and which were not visited by insects.

**1. Estimation of number of anthers per flower**

Numbers of stamens per flower were recorded for all flowers sampled, and are given in Table S3. This number was constant across flowers within a species for most taxa sampled.

**2. Estimation of number of pollen grains per anther**

(i) Collection of pollen from anthers. To obtain a measurable volume of pollen for estimation of volume per flower, we pooled pollen from multiple anthers, with larger numbers from species whose flowers have small anthers and/or small numbers of stamens per flower. Thus to obtain a measurable volume, we combined the pollen from all of the stamens of 20 flowers of *Sisymbrium officinale*, 20 stamens from 4 flowers of *Echium vulgare*, all of the stamens from 60 single flowers of *Daucus carota*, but only 15 stamens from 1 flower of *Papaver rhoeas*. When examination with a hand lens showed the anthers to be fully dehisced, we carefully transferred the required number of anthers to a 1.5ml eppendorf tube containing 1.0 ml of 70% ethanol.

Pollen was extracted from samples in a series of rinses in which the pooled anthers were ultrasonicated (Dawe sonicleaner, UK) in 70% ethanol, as follows:

First rinse. The sample eppendorf was vortex mixed for 30 seconds, and ultrasonicated for 10 minutes. The sample was then vortexed for another 30 seconds. As much of the 70% ethanol containing pollen was transferred to a clean 1.5ml eppendorf tube.

Second rinse. After adding 300µl of 70% ethanol, we repeated the procedure for the first rinse, above, transfering the 70% ethanol containing pollen to the same 1.5ml eppendorf.

Third rinse. After adding 200µl of 70% ethanol, we repeated the procedure for the first rinse, above, transfering the 70% ethanol containing pollen to the same 1.5ml eppendorf.

The anthers were checked under a microscope to confirm liberation of all of the pollen. In a very small number of cases, a fourth rinse with 200µl 70% ethanol was required. The eppendorf for each sample was centrifuged for 10 minutes at 13 000 rpm to pellet the pollen. As much of the supernatant as possible was then removed, taking care not to disturb the pellet. The pellet was air dried for 2h in a 60°C oven to remove the remainder.

(ii) Estimation of numbers of pollen grains per anther. For each sample, the pelleted pollen grains were first resuspended (ultrasonicate for 10 min, shake for 30s) in a recorded volume of 70% ethanol (50 to 200 µl, depending on the size of the pellet). We then counted the number of pollen grains in three 10µl aliquots of the pollen suspension using a haemocytometer. The haemocytometer was cleaned and the pollen suspension shaken for 30 seconds before the removal of each subsequent 10µl aliquot. For each aliquot, we sampled enough of the haemocytometer volume to reach counts of *ca.* 500 pollen grains. We calculated the mean number of pollen grains per µl across the three aliquots for each sample, and multiplied this by the total sample volume (either 50 to 200µl) to reach an estimate of the total number of pollen grains in the sample. This value divided by the number of anthers pooled in the sample gives the mean number of pollen grains per anther.

**3. Estimating the volume of a pollen grain**

We measured the major and minor axes of 10 pollen grains per sample (as shown below) using an eyepiece graticule, most often at 40x magnification. Pollen grain volume was estimated using the formula for a three dimensional ellipsoid,

*V*=4/3 *πAB^2^*, where *A* is half of the longest (major) axis of the pollen grain, and *B*  is half the minor axis.

**
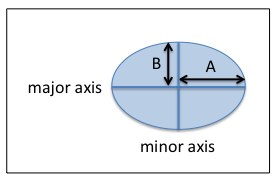
**
