# Supplementary material for: Food for Pollinators: Quantifying the Nectar and Pollen Resources of Urban Flower Meadows
Source: PLoS One. 2016 Jun 24;11(6):e0158117. doi: 10.1371/journal.pone.0158117 (PMC4920406; doi:10.1371/journal.pone.0158117)
Supplement: S2 Table — Study site information for each of the four cities (Bristol, Edinburgh, Leeds and Reading). Site location information includes the location name (most often a park or school), street name location, and UK vice county (VC) name and number. The column ‘Block’ refers to the location of each site within 5 geographically grouped sampling blocks in each city. (DOCX) [file pone.0158117.s012.docx]

**Table S2**. Study site information for each of the four cities (Bristol, Edinburgh, Leeds and Reading). Site location information includes the location name (most often a park or school), street name location, and vice county (VC) name and number. The column ‘Block’ refers to the location of each site within 5 geographically grouped sampling blocks in each city. Treatments are Annual 1 year (A1), Annual for two years (A2), Perennial (PL) and amenity grassland control (CL). Sites that failed due to management problems, and which were excluded from statistical analyses, are indicated by the word ‘fail’ in the Treatment column.

| **Treatment** | **Meadow Name** | **City** | **OS Grid Reference** | **VC Name** | **VC Number** | **Block** | **Street Site Name** |
| --- | --- | --- | --- | --- | --- | --- | --- |
| A1 | Castle Park | Bristol | ST591731 | West Gloucestershire | 34 | Central Bristol | Broad Weir |
| A2 | City Academy | Bristol | ST611734 | West Gloucestershire | 34 | Central Bristol | The City Academy, Russell Town Avenue |
| PL | Stoke Bishop Halls | Bristol | ST566758 | West Gloucestershire | 34 | Central Bristol | University of Bristol Stoke Bishop Halls Campus |
| CL | Royal Fort Gardens | Bristol | ST582733 | West Gloucestershire | 34 | Central Bristol | Tyndall Avenue |
| A1 | Ridgeway Playing Fields | Bristol | ST630749 | West Gloucestershire | 34 | East Bristol | Foundary Lane |
| A2 | St George Park | Bristol | ST622737 | West Gloucestershire | 34 | East Bristol | St George Park |
| PL | Beacon Rise Primary School | Bristol | ST648727 | West Gloucestershire | 34 | East Bristol | Hanham Road |
| CL | Rodney Road | Bristol | ST635739 | West Gloucestershire | 34 | East Bristol | Rodney Road Playing Field |
| A1 | Little Mead Primary School | Bristol | ST580783 | West Gloucestershire | 34 | North Bristol | Gosforth Road |
| A2 | Henleaze Junior School | Bristol | ST582763 | West Gloucestershire | 34 | North Bristol | Park Grove |
| PL | Horfield Common | Bristol | ST591767 | West Gloucestershire | 34 | North Bristol | Kellaway Avenue |
| CL | Monks Park | Bristol | ST594780 | West Gloucestershire | 34 | North Bristol | Lyddington Road |
| A1 | Greenfield Primary School | Bristol | ST583693 | North Somerset | 6 | South Bristol | Novers Lane |
| A2 | Merchants Academy | Bristol | ST576682 | North Somerset | 6 | South Bristol | Gatehouse Avenue |
| PL | Hengrove Park | Bristol | ST597688 | North Somerset | 6 | South Bristol | Bamfield |
| CL | Briery Leaze Road | Bristol | ST601681 | North Somerset | 6 | South Bristol | Bamfield |
| A1 | Hengrove Farm | Bristol | ST603695 | North Somerset | 6 | South Central | Walsh Avenue |
| A2 | Gores Marsh | Bristol | ST570709 | North Somerset | 6 | South Central | Winterstoke Road |
| PL Fail | Victoria Park | Bristol | ST593715 | West Gloucestershire | 34 | South Central | Bedminster |
| CL | Salcombe Road | Bristol | ST601702 | North Somerset | 6 | South Central | Salcombe Road Recreation Ground |
| A1 | Jewel Park | Edinburgh | NT302723 | Midlothian | 83 | East Edinburgh | Duddingston Park South |
| A2 | Cairntows Park | Edinburgh | NT286716 | Midlothian | 83 | East Edinburgh | Peffer Bank |
| CL | Morgan Playing Fields | Edinburgh | NT281717 | Midlothian | 83 | East Edinburgh | Peffermill Road |
| PL | Joppa Quarry Park | Edinburgh | NT313731 | Midlothian | 83 | East Edinburgh | Milton Road East |
| A1 | Montgomery Street | Edinburgh | NT268746 | Midlothian | 83 | North Edinburgh | Montgomery Street Park |
| A2 | West Pilton Park | Edinburgh | NT223758 | Midlothian | 83 | Northwest Edinburgh | West Pilton Avenue |
| CL | Leith Links | Edinburgh | NT274758 | Midlothian | 83 | North Edinburgh | Leigh Links West |
| PL | Pilrig Park | Edinburgh | NT262756 | Midlothian | 83 | North Edinburgh | Pilrig Street |
| A1 | Drumbrae Drive | Edinburgh | NT201744 | Midlothian | 83 | Northwest Edinburgh | Drum Brae Drive |
| A2 | Davidson's Mains School | Edinburgh | NT209752 | Midlothian | 83 | Northwest Edinburgh | Corbiehill Road |
| CL | Orchard Brae | Edinburgh | NT229742 | Midlothian | 83 | Northwest Edinburgh | Queensferry Road |
| PL | St Marks Park | Edinburgh | NT256756 | Midlothian | 83 | Northwest Edinburgh | Warriston Road |
| A1 | Firrhill School | Edinburgh | NT227698 | Midlothian | 83 | South Edinburgh | Oxgangs Road North |
| A2 | Inch Park | Edinburgh | NT274707 | Midlothian | 83 | South Edinburgh | Old Dalkeith Road |
| CL | Morningside Park | Edinburgh | NT240707 | Midlothian | 83 | South Edinburgh | Morningside Drive |
| PL | St Katharine's Park | Edinburgh | NT273688 | Midlothian | 83 | South Edinburgh | Liberton Gardens |
| A1 | Hailes Quarry Park | Edinburgh | NT207703 | Midlothian | 83 | Southwest Edinburgh | Dumbryden Drive |
| A2 | Sighthill Park | Edinburgh | NT197712 | Midlothian | 83 | Southwest Edinburgh | Broomhouse Road |
| CL | Union Park | Edinburgh | NT203720 | Midlothian | 83 | Southwest Edinburgh | Carrick Knowe Drive |
| PL Fail | Saughton Park | Edinburgh | NT221718 | Midlothian | 83 | Southwest Edinburgh | Balgreen Road |
| A1 | Seacroft Ring Road | Leeds | SE354371 | Mid-west Yorkshire | 64 | East Leeds | Seacroft Ring Road |
| A2 | East End Park | Leeds | SE325333 | Mid-west Yorkshire | 64 | East Leeds | Victoria Avenue |
| CL | Harehills Park | Leeds | SE329349 | Mid-west Yorkshire | 64 | East Leeds | Coldcotes Avenue |
| PL | Ebor Gardens | Leeds | SE317339 | Mid-west Yorkshire | 64 | East Leeds | Torre Road |
| A1 | Chapel Allerton Park | Leeds | SE307374 | Mid-west Yorkshire | 64 | North Leeds | Chapel Allerton Park |
| A2 | King Lane | Leeds | SE296391 | Mid-west Yorkshire | 64 | North Leeds | King Lane, Moortown |
| CL | Potternewton Park | Leeds | SE314360 | Mid-west Yorkshire | 64 | North Leeds | Harehills Lane |
| PL | Scott Hall | Leeds | SE300364 | Mid-west Yorkshire | 64 | North Leeds | Potternewton Lane |
| A1 | West Park | Leeds | SE264373 | Mid-west Yorkshire | 64 | NW Leeds | North Parade |
| A2 | Stanningley Park | Leeds | SE229350 | South-west Yorkshire | 63 | NW Leeds | Stanningley Park, Leeds |
| CL | Beckett Park | Leeds | SE268366 | Mid-west Yorkshire | 64 | NW Leeds | Beckett Park, Headingley |
| PL | Stanhope Rec/Drury Lane | Leeds | SE241379 | Mid-west Yorkshire | 64 | NW Leeds | Drury Avenue |
| A1 | Hunslet Moor | Leeds | SE304315 | South-west Yorkshire | 63 | South Leeds | Burton Row |
| A2 | Middleton Park | Leeds | SE299285 | South-west Yorkshire | 63 | South Leeds | Middleton Park |
| CL | Bow Street Recreation Ground | Leeds | SE311330 | Mid-west Yorkshire | 63 | South Leeds | Bow Street |
| PL | Cross Flatts | Leeds | SE295305 | South-west Yorkshire | 63 | South Leeds | Dewsbury Road |
| A1 | Queen's Park | Leeds | SE217332 | South-west Yorkshire | 63 | West Leeds | Queen's Park |
| A2 | Armley Park | Leeds | SE269339 | South-west Yorkshire | 63 | West Leeds | Stanningley Road |
| CL | Western Flatts Park | Leeds | SE264323 | South-west Yorkshire | 63 | West Leeds | Green Hill Lane |
| PL | Burley Park | Leeds | SE279346 | Mid-west Yorkshire | 64 | West Leeds | Burley Road |
| A1 | Palmer Park | Reading | SU710724 | Berkshire | 22 | Central Reading | Wokingham Road |
| A2 | Christchurch Meadows | Reading | SU718742 | Oxfordshire | 23 | Central Reading | George Street |
| CL | Kings Meadow | Reading | SU722739 | Berkshire | 22 | Central Reading | Napier Road |
| PL | Portman Road East | Reading | SU700738 | Berkshire | 22 | Central Reading | Portman Road |
| A1 Fail | Thames Promenade | Reading | SU708745 | Berkshire | 22 | North Reading | Thames Promenade |
| A2 | Caversham Crematorium | Reading | SU723757 | Oxfordshire | 23 | North Reading | All Hallows Road |
| CL | Thames Promenade control | Reading | SU702748 | Berkshire | 22 | North Reading | Thames Path |
| PL | Westfield Road Recreation Ground | Reading | SU716747 | Oxfordshire | 23 | North Reading | Falkland Road |
| A1 | Whitley Wood Recreation Ground | Reading | SU716698 | Berkshire | 22 | South Reading | Basingstoke Road |
| A2 | Rabsons Recreation Ground | Reading | SU724701 | Berkshire | 22 | South Reading | Northumberland Avenue |
| CL | Longbarn Lane Recreation Ground | Reading | SU720712 | Berkshire | 22 | South Reading | Long Barn Lane |
| PL | Cintra Park | Reading | SU722718 | Berkshire | 22 | South Reading | Northumberland Avenue |
| A1 | Coley Recreation Ground | Reading | SU410724 | Berkshire | 22 | South West Reading | St Saviour's Road |
| A2 | Prospect Park West | Reading | SU685723 | Berkshire | 22 | South West Reading | Bath Road |
| CL | Courage Park | Reading | SU700722 | Berkshire | 22 | South West Reading | Edenham Crescent |
| PL | Prospect Park East | Reading | SU692729 | Berkshire | 22 | South West Reading | Tilehurst Road |
| A1 Fail | Portman Road West | Reading | SU690741 | Berkshire | 22 | West Reading | Portman Road |
| A2 | Victoria Recreation Ground | Reading | SU671741 | Berkshire | 22 | West Reading | Armour Road |
| CL | Wilson Road Playing Fields | Reading | SU691734 | Berkshire | 22 | West Reading | Tofrek Terrace |
| PL | Meadway Copse Recreation Ground | Reading | SU675732 | Berkshire | 22 | West Reading | Church End Lane |
