# Supplementary material for: Food for Pollinators: Quantifying the Nectar and Pollen Resources of Urban Flower Meadows
Source: PLoS One. 2016 Jun 24;11(6):e0158117. doi: 10.1371/journal.pone.0158117 (PMC4920406; doi:10.1371/journal.pone.0158117)
Supplement: S4 Table — The species listed in each table comprise those with an individual p ≤ 0.05 or the set of species contributing cumulatively to 50% of the explained variance, whichever was greater. The status of each species is shown as sown (i.e. in a seed mix) or weed. The right hand column shows the direction of trends in abundance of each species across cities. (DOCX) [file pone.0158117.s014.docx]

**Table S4**. Univariate analysis outputs of mvabund, showing species contributing most significantly to variation in meadow composition (incorporating both presence absence and relative abundance of floral units across surveys). The species listed in each table comprise those with an individual p ≤ 0.05 or the set of species contributing cumulatively to 50% of the explained variance, whichever was greater. The status of each species is shown as sown (ie in a seed mix) or weed. The right hand column shows the direction of trends in abundance of each species across cities.

A. Plant species contributing to differences between perennial meadows across cities

| **Perennial meadow species** | **sown / weed** | **deviance explained** | **p** | **% of overall deviance** | **cumulative % deviance** | **cross-city patterns of abundance** |
| --- | --- | --- | --- | --- | --- | --- |
| *Achillea millefolium* | sown | 32.902 | 0.001 | 4.7 | 5 | ED > LD > RD > BR |
| *Stellaria media* | weed | 27.765 | 0.001 | 3.9 | 9 | ED > RD > LD |
| *Crepis capillaris* | weed | 27.190 | 0.001 | 3.8 | 12 | RD > |
| *Epilobium spp* | weed | 25.885 | 0.001 | 3.7 | 16 | ED > BR >LD |
| *Capsella bursa_pastoris* | weed | 24.462 | 0.001 | 3.5 | 20 | ED > RD |
| *Polygonum aviculare* | weed | 21.421 | 0.003 | 3.0 | 23 | ED > RD |
| *Malva moschata* | sown | 21.059 | 0.003 | 3.0 | 26 | LD > |
| *Sisymbrium officinale* | weed | 21.050 | 0.003 | 3.0 | 29 | ED > RD |
| *Reseda lutea* | sown | 20.054 | 0.005 | 2.8 | 31 | ED > BR |
| *Medicago lupulina* | weed | 18.091 | 0.010 | 2.6 | 34 | RD > |
| *Malva spp* | weed | 17.718 | 0.010 | 2.5 | 36 | BR > RD > ED |
| *Hypochaeris radicata* | weed | 17.684 | 0.010 | 2.5 | 39 | RD > BR |
| *Pulicaria dysenterica* | sown | 17.296 | 0.011 | 2.4 | 41 | BR > LD |
| *Scorzoneroides autumnalis* | weed | 15.959 | 0.021 | 2.3 | 44 | RD > LD > BR |
| *Plantago major* | weed | 15.737 | 0.022 | 2.2 | 46 | LD > BR |
| *Taraxacum agg* | weed | 15.583 | 0.026 | 2.2 | 48 | ED > |
| *Stellaria graminea* | weed | 15.330 | 0.027 | 2.2 | 50 | ED > |
| *Origanum vulgare* | sown | 15.022 | 0.031 | 2.1 | 52 | BR > LD > RD |
| *Echium vulgare* | sown | 14.720 | 0.031 | 2.1 | 54 | ED > LD > BR > RD |
| *Knautia arvensis* | sown | 14.287 | 0.034 | 2.0 | 56 | BR > ED |

B. Plant species contributing to differences between annual meadows across cities

| **Annual meadow species** | **sown / weed** | **deviance explained** | **p** | **% of overall deviance** | **cumulative % deviance** | **cross-city patterns in abundance** |
| --- | --- | --- | --- | --- | --- | --- |
| *Lobularia maritima* | sown | 96.948 | 0.001 | 8.3 | 8 | BR > ED > LD > RD |
| *Coreopsis* spp. | weed | 52.412 | 0.001 | 4.5 | 13 | BR > LD > ED |
| *Polygonum aviculare* | weed | 52.358 | 0.001 | 4.5 | 17 | ED > LD |
| *Malcolmia maritima* | sown | 52.193 | 0.001 | 4.5 | 22 | BR > RD > ED > LD |
| *Cosmos bipinnatus* | sown | 48.088 | 0.001 | 4.1 | 26 | LD > BR > RD > ED |
| *Scorzoneroides autumnalis* | weed | 43.259 | 0.001 | 3.7 | 30 | LD > RD > BR |
| *Achillea millefolium* | weed | 40.679 | 0.001 | 3.5 | 33 | LD > RD |
| *Crepis capillaris* | weed | 39.369 | 0.001 | 3.4 | 36 | RD > |
| *Epilobium* spp. | weed | 37.712 | 0.001 | 3.2 | 40 | ED > RD > BR |
| *Veronica agrestis polita persica* | weed | 35.063 | 0.001 | 3.0 | 43 | RD > BR > LD |
| *Linum grandiflorum* | sown | 31.851 | 0.001 | 2.7 | 45 | LD > BR > RD > ED |
| *Stellaria media* | weed | 30.339 | 0.001 | 2.6 | 48 | ED > LD > BR |
| *Sonchus oleraceus* | weed | 29.990 | 0.001 | 2.6 | 51 | RD > BR > ED > LD |
| *Thelesperma burridgeanum* | sown | 24.542 | 0.001 | 2.1 | 53 | LD > BR > RD > ED |
| *Lapsana communis* | weed | 24.212 | 0.001 | 2.1 | 55 | RD > |
| *Plantago major* | weed | 24.189 | 0.001 | 2.1 | 57 | LD > BR |
| *Euphorbia helioscopia* | weed | 22.276 | 0.003 | 1.9 | 59 | RD > |
| *Centaurea cyanus* | sown | 21.978 | 0.003 | 1.9 | 61 | LD > ED > BR > RD |
| *Anagallis arvensis* | weed | 20.223 | 0.003 | 1.7 | 62 | RD > BR |
| *Matricaria discoidea* | weed | 20.199 | 0.003 | 1.7 | 64 | ED > LD |
| *Convolvulus arvensis* | weed | 18.332 | 0.009 | 1.6 | 66 | RD > |
| *Eschscholzia californica* | sown | 17.592 | 0.011 | 1.5 | 67 | LD > BR > ED > RD |
| *Sonchus asper* | weed | 17.289 | 0.014 | 1.5 | 69 | ED > BR > RD > LD |
| *Lactuca serriola* | weed | 16.740 | 0.017 | 1.4 | 70 | RD > BR |
| *Calendula officinalis* | sown | 16.119 | 0.022 | 1.4 | 72 | BR > LD > RD > ED |
| *Cerastium fontanum* | weed | 15.201 | 0.033 | 1.3 | 73 | ED > |
| *Persicaria maculosa* | weed | 15.002 | 0.036 | 1.3 | 74 | RD > LD |
| *Fumaria* spp. | weed | 14.808 | 0.036 | 1.3 | 75 | ED > RD |
| *Tripleurospermum inodorum* | weed | 14.216 | 0.042 | 1.2 | 77 | RD > LD |

C. Plant species contributing to differences between annual meadow treatments (i.e. A1 vs. A2) across cities

| **Annual meadow species** | **sown / weed** | **deviance explained** | **p** | **% of overall deviance** | **cumulative % deviance** | **cross-city patterns of abundance** |
| --- | --- | --- | --- | --- | --- | --- |
| *Veronica agrestis/polita/persica* | weed | 11.026 | 0.045 | 7.9 | 8 | A2 > A1 |
| *Epilobium* spp. | weed | 9.046 | 0.107 | 6.5 | 14 | A2 > A1 |
| *Stellaria media* | weed | 8.881 | 0.118 | 6.4 | 21 | A2 > A1 |
| *Ranunculus repens* | weed | 7.826 | 0.200 | 5.6 | 26 | A2 > A1 |
| *Borago officinalis* | weed | 5.473 | 0.629 | 3.9 | 30 | A2 > A1 |
| *Lobularia maritima* | sown | 4.722 | 0.803 | 3.4 | 34 | A1 > A2 |
| *Viola* spp. | weed | 4.372 | 0.845 | 3.1 | 37 | A1 > A2 |
| *Lamium album* | weed | 4.092 | 0.922 | 2.9 | 40 | A2 > A1 |
| *Prunella vulgaris* | weed | 4.085 | 0.922 | 2.9 | 43 | A2 > A1 |
| *Helminthotheca echioides* | weed | 3.881 | 0.943 | 2.8 | 45 | A2 > A1 |
| *Achillea millefolium* | weed | 3.808 | 0.945 | 2.7 | 48 | A2 > A1 |

D. Plant species contributing most to the interaction between annual meadow treatment (i.e. A1 vs. A2) and city

| **Annual meadow species** | **sown / weed** | **deviance of Treatment:City** | **p (> deviance of Treatment:City)** | **% of overall deviance** | **cumulative % deviance** |
| --- | --- | --- | --- | --- | --- |
| *Calendula officinalis* | sown | 25.789 | 0.001 | 7.6 | 8 |
| *Polygonum aviculare* | weed | 22.755 | 0.001 | 6.7 | 14 |
| *Achillea millefolium* | weed | 18.995 | 0.002 | 5.6 | 20 |
| *Ranunculus repens* | weed | 18.582 | 0.002 | 5.5 | 25 |
| *Persicaria maculosa* | weed | 16.986 | 0.010 | 5.0 | 30 |
| *Sisymbrium officinale* | weed | 16.048 | 0.017 | 4.7 | 35 |
| *Plantago lanceolata* | weed | 14.002 | 0.043 | 4.1 | 39 |
| *Epilobium* spp. | weed | 12.331 | 0.103 | 3.6 | 43 |
| *Thelesperma burridgeanum* | sown | 11.946 | 0.124 | 3.5 | 46 |
| *Lamium purpureum* | weed | 10.627 | 0.207 | 3.1 | 49 |
